# Prolonged grief disorder symptomology in three African countries: A network analysis and comparison

**DOI:** 10.1017/gmh.2024.54

**Published:** 2024-04-29

**Authors:** Martin Robinson, Chérie Armour, Yafit Levin

**Affiliations:** 1 Research Centre for Stress Trauma and Related Conditions (STARC), School of Psychology, Queen’s University Belfast, Northern Ireland, UK; 2 Department of Social Work and Education, Ariel University, Ariel Israel

**Keywords:** ICD-11, Prolonged grief disorder, Network analysis, Network comparison, Cross-culture

## Abstract

**Background:**

Bereavement is a globally prevalent life stressor, but in some instances, it may be followed by a persistent condition of grief and distress, codified within the 11th edition of the International Classification of Diseases (ICD-11) as prolonged grief disorder (PGD). Network analysis provides a valuable framework for understanding psychological disorders at a nuanced symptom-based level.

**Aim:**

This study novelly explores the network structure of ICD-11 PGD symptomology in a non-Western sample and assesses the replication of this across three African country sub-samples in these data.

**Methodology:**

Network models were estimated using the “Inventory of Complicated Grief-Revised” in a sample of trauma-exposed individuals who experienced bereavement throughout life (*N* = 1,554) from three African countries (Ghana, *n* = 290; Kenya, *n* = 619; Nigeria, *n* = 645). These networks were statistically evaluated using the network comparison test.

**Results:**

It was found that “Feelings of Loss” and “Difficulty moving on” were the most central symptoms in the combined sample network. These findings were largely consistent for the Ghana and Nigeria sub-samples, however, network structure differences were noted in the Kenya sub-sample.

**Conclusion:**

The identified PGD network highlights particular indicators and associations across three African samples. Implications for the assessment and treatment of PGD in these cultural contexts warrant consideration.

## Graphical abstract

**Figure:**
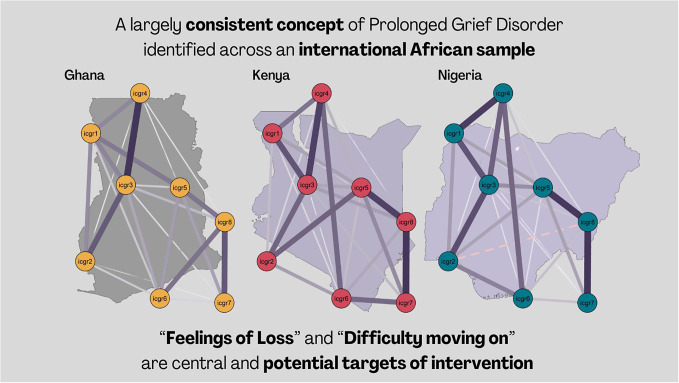

## Impact statement

Bereavement and loss, a nearly ubiquitous stressor, may lead to prolonged grief disorder (PGD) in a minority of individuals, marked by an extended period of yearning, preoccupation and emotional pain. This study identified “Feelings of Loss” and “Difficulty moving on” as critical factors influencing PGD, suggesting these as potentially valuable targets for intervention to alleviate disordered grief. Indeed, by addressing these factors, interventions may reduce distress linked to PGD through the prevention of the cascading effects within the symptom network. This study identified consistency in symptom networks across sub-samples drawn from three African nations, emphasising the global relevance of PGD. However, some divergence was observed when comparing the Kenyan sample to those from Nigeria and Ghana. This highlights the importance of recognising cultural nuances in the assessment of PGD, and the formulation of treatment approaches. The evidence supports the view that the criteria for PGD outlined in ICD-11 are relevant on a global scale, emphasising the need for psychological interventions that are sensitive to cultural considerations. Addressing “Feelings of Loss” and “Difficulty moving on” within psychological intervention and bereavement support may, however, be considered internationally relevant to enhance the effectiveness of psychological support for those grappling with prolonged grief.

## Introduction

Bereavement, the death of a loved one, is a common and universal stressor, often leading to a grieving process marked by loss, yearning and sadness (Hall, 2014; Hilberdink et al., 2023). While most people adapt over time, some experience disordered chronic and complex grief reactions persisting beyond typical cultural bereavement periods (Bonanno et al., 2002).

This experience is codified as prolonged grief disorder (PGD) in the 11th edition of the “International Classification of Diseases” (ICD-11), characterised primarily by persistent and pervasive *longing* and/or *preoccupation* with the deceased accompanied by intense emotional pain, that is, feelings related to grief which may cause significant distress such as sadness, guilt, anger, bitterness, or feeling one has lost a part of themselves (see Eisma et al., 2020; WHO, 2023). Within PGD, a pivotal criterion involves significant challenges persisting for a duration that deviates from the normative grief process, usually 6 months or longer (WHO, 2023). Several different statistical methods have been used to distinguish current PGD criteria, including confirmatory factor analysis (Boelen et al., 2008) and latent class analysis (Boelen et al., 2016).

The World Health Organisation’s (WHO) revisions in ICD-11 aim to streamline stressor-related disorders for broader global use (Maercker and Eberle, 2022). PGD is uniquely challenging due to its cultural specificity compared to other disorders in this category (Maercker and Eberle, 2022). Although PGD’s measurements have been validated widely in Western settings (Prigerson et al., 2009; Prigerson et al., 2021), it is important to recognise that various cultural beliefs about death can extend grief, which does not necessarily signal a disorder.

The current iteration of the Diagnostic and Statistical Manual (DSM-5-TR; APA, 2022) likewise contains a categorisation of PGD. In both manuals, the experience of bereavement and distress is characterised by preoccupation and longing for the deceased (Eisma, 2023). Likewise, both require that indicators of grief-related distress exceed social, cultural or religious norms established as part of cultural expectations (Eisma, 2023). Indeed, previous research has highlighted cultural differences in the endorsement of PGD symptomology, ultimately leading to heterogeneity in expression and assessment (see Stelzer et al., 2019; Eisma, 2023), which are attributed to heterogeneity in grief practices and individual interpretation of distress.

In African cultures, where family and community ties are of great importance, the loss of a loved one may be more likely to result in prolonged distress (McCarthy et al., 2023). This underscores the need to validate grief disorder frameworks to reflect cultural differences and contextual influences. Indeed, PGD involves persistent yearning for the deceased, which may be analogous to expression through rituals, like in Sub-Saharan cultures where communities and families communicate with the deceased for guidance during public grieving (Lechner-Meichsner and Comtesse, 2022). Such prolonged fixation on the deceased may be adaptive, facilitating grief processing rather than an indicator of disordered grief or distress. Conversely, the Tiv people of Nigeria follow prescriptive traditional practices that, if interrupted or not applied due to the experience of a “bad death,” for example, the death of a child or violent circumstances, maybe a catalyst driving prolonged distress and disordered grief reactions (Chukwuorji et al., 2018).

PGD is distinguished from typical acute grief, characterised by resolution within 6 months and by symptoms like sadness, sleep issues and emotional reactivity (Bonanno and Malgaroli, 2020; Mughal et al., 2022). Typical grief expressions, however, vary culturally (Hilberdink et al., 2023). For example, some African cultures view grief differently, with individuals comforted by the belief in the continued presence of the deceased’s spirit (Njue et al., 2015). Persistent feelings of grief and yearning may therefore be less distressing to individuals based on cultural expectations and norms, contributing to findings that PGD prevalence is elevated in westernised contexts compared to the relative symptom burden reported in the global south (Ben-Ezra et al., 2020; Hilberdink et al., 2023). PGD has context-specific considerations for symptoms and functioning (Maciejewski et al., 2016), necessitating culturally sensitive diagnostic criteria (Maercker and Eberle, 2022). Differentiating PGD from depression and PTSD requires an understanding of its core symptoms and the impact on those experiencing symptomatology (Prigerson et al., 2009; Smith and Ehlers, 2021). This is complemented by conceptual advances towards analysing individual symptoms and their interrelations rather than relying solely on aggregate scores or diagnostic checklists (Fried, 2017).

Network Analysis in psychopathology provides such an opportunity, suggesting that disorders stem from complex symptom interactions, with central, highly connected symptoms driving the development and persistence of mental illness (Borsboom and Cramer, 2013). Indeed, previous research has shown that the use of Network approaches may provide more valuable insight into the direct association between individual symptoms of grief and distress (Fried et al., 2015). Researchers have previously applied this method to the study of disordered grief (see Robinaugh et al., 2014, 2016); finding that an independent indicator of *emotional pain* is the most central, suggesting this may serve a pivotal role in a positive feedback loop between symptoms, for example: yearning → emotional pain → thoughts about the deceased → yearning, leading to their exacerbation and maintenance over time. This is interesting because in these investigations based on the Changing Lives of Older Couples study data emotional pain was not the indicator most consistently endorsed (rather it was yearning), however, emotional pain was the indicator that activated the pathological manifestation of PGD (Robinaugh et al., 2014, 2016).

Maccallum et al. (2017) explored the network structure of PGD among Danish adults bereaved of a spouse, similarly finding a strong link between “yearning” and “emotional pain.” Further analysis revealed “meaninglessness,” “emptiness” and “numbness" as central to symptom networks a year post-bereavement, indicating their role in sustaining disordered grief per PGD criteria (Maccallum et al., 2023). These results were noted to echo that of Robinaugh et al. (2016); suggesting the importance of emotional pain and profound feelings of loss in parts of one’s self in the manifesting and expression of disordered grief. This is extended by the work of Maccallum and Bryant (2020) highlighting that in a network of PGD symptomology and indicators of quality of life: feelings of meaninglessness, confusion and like a part of yourself has died were most associated with poorer perceived quality of life. These difficulties, that is, longing and emotional pain, have been identified as highly influential in networks of disordered grief and drivers of comorbidity with traumatic stress and depression (Malgaroli et al., 2018), suggesting the need to better understand and address these in relation to disordered grief.

While emotional pain and feelings of emptiness are not considered ‘hallmarks’ for PGD according to ICD-11 criteria (WHO, 2023), they may be considered characteristic of this disorder; and in turn, driving symptom expression and change in the network (Robinaugh et al., 2016; Castro et al., 2019). Indeed, it is held in Network Analyses that the most central nodes are not necessarily those that are the hallmarks of the disorder (McNally, 2016; Castro et al., 2019). The *most* central symptoms may, therefore, be considered particularly important targets for intervention, serving to deactivate networks of distress as alleviating symptom distress is argued to have a cascading effect as those most central or influential symptoms are likely to decrease others connected to them (Castro et al., 2019). Robinaugh et al. (2022, 2014) have argued in favour of the network approach to understanding psychopathology as this does not adhere to the same prescription of the latent variable model approach, suggesting difficulties arise from an unobserved common cause but are instead causally linked to each other. The current study complements and extends the prior evidence presented, contributing to further investigation of PGD using data from the global south, across three African countries.

Based on the extant literature, the current study sought to investigate the following hypotheses utilising network analysis methodology:

H1. In line with previous network evidence; grief reactions indicative of *emotional pain* and *feelings of loss* will present as the most central in the network of PGD symptoms.

H2. The symptom network will remain stable in a total sample network comprising data from all three countries, and replicate when data are parsed to country sub-samples (i.e., for samples from each nation).

## Methodology

### Data and sample

This study analysed a sub-sample of self-report survey data drawn from three African countries; Ghana, Kenya and Nigeria (total *N* = 2,524 participants). Data were collected using panel methodology, obtaining samples approximately representative in terms of age and gender in these three countries. Participants were considered eligible if they had residential status in any of the three aforementioned countries, were aged 18 or older, and possessed English proficiency to complete survey measures. Further information on this sample and procedure is provided by Ben-Ezra et al. (2020).

## Measures

*Experience of bereavement throughout life*, that is, the death of a loved one experienced at any point in one’s lifetime, was measured (“*Ever happened to you: Death of a loved one”*), and in the previous year (“*Did you experience loss (death of a loved one) in the past year?*”). These items were rated dichotomously, the former was used to screen participants for eligibility in these analyses. Respondents were also asked to self-report the number of bereavement experiences they had in the previous 5 years (“*How many people did you lose (death of loved ones) in the last 5 years?*”).

*Prolonged Grief Disorder symptomology* was measured using the Inventory of Complicated Grief-Revised (ICGR; Prigerson and Jacobs, 2001), an abbreviated and validated version of the original Inventory of Complicated Grief (Prigerson et al., 1995). The ICGR consists of seven items related to ICD-11 PGD criteria, plus a rating of functional impairment experienced in relation to these difficulties. Participants are asked to rate difficulty experienced on a 5-point Likert scale from 1 “*Almost never*” to 5 “*Always*” (see Table 1). Although the ICGR was developed before the most recent classifications of PGD, the current study applied an algorithmic assessment of disorder grief symptomology in line with ICD-11 proposals (WHO, 2023). This method of screening assessment has been used in previous research using general measures of disordered grief, specifically the ICGR, and applying current criteria for ICD-11 PGD pathology (Killikelly and Maercker, 2023; Bagcaz and Kilic, 2023; Ben-Ezra et al., 2020).

**Table 1.** Item codes and labels for inventory of complicated grief items use in primary analyses

Item code	Brief label	Item
ICGR1	“Preoccupation”	I think about this person so much that it’s hard for me to do the things I normally do
ICGR2	“Longing”	I feel myself longing and yearning for the deceased person
ICGR3	“Feelings of Loss”	I feel as if a part of me died
ICGR4	“Disbelief”	I feel disbelief over the deceased person’s death.
ICGR5	“Difficulty moving on”	Ever since the deceased person died, I find it difficult to move on with my life
ICGR6	“Bitterness”	I am bitter over the deceased person’s death.
ICGR7	“Guilt”	I feel that it is unfair that I should live when the deceased person died
ICGR8	“Impairment”	I believe that my grief has resulted in impairment in my social, occupational or other areas of functioning

Probable PGD was screened in line with procedures of those previous studies (see Ben-Ezra et al., 2020; Bagcaz and Kilic, 2023), requiring endorsement of *Longing* (ICGR1) or *Preoccupation* (ICGR2), plus three additional grief reactions indicative of emotional pain (ICGR3:ICGR7) to a significant degree (4 “*Almost always*” or 5 “*Always*”). Extant evidence suggests disordered grief is well represented by a unidimensional construct measured by the ICGR (Maciejewski et al., 2016; Schakowski et al., 2023), thus total scores were also compared within descriptive analyses (Range: 7–35). Scale internal reliability was favourable in the total study sample comprising data from all counties (α = .903), and country sub-samples (Ghana, α = .912; Kenya, α = .922; Nigeria, α = .905).

## Procedure

### Descriptive and comparative statistics

Descriptive and summary statistics were generated using the “*gtsummary*” package in R (Sjoberg et al., 2021). Comparisons between groups were conducted using “*rstatix*” (Kassambara, 2023) and visualised using “*ggplot2*” (Wickham, 2016). Differences between groups were tested using Chi-square test for categorical variables and Kruskal-Wallis test for ordinal/continuous data. Where statistically significant differences were observed between groups, analyses were supplemented using Dunn’s pairwise comparisons or Wilcoxon tests with Bonferroni correction for categorical and continuous data, respectively (Mangiafico, 2023).

### Network estimation

Networks were estimated using the R package “*bootnet”* (Epskamp et al., 2018), visualised using “*qgraph*” (Epskamp et al., 2012) and compared using “*NetworkTools”* (Jones, 2022). In this output, each item on the ICGR is represented by a node, and the association between items is represented by an edge, a weight line connecting nodes. Network estimation used a Gaussian graphical model based on *Spearman’s Correlation Coefficients*, accounting for non-normal distribution of these data. In line with current guidance (Epskamp and Fried, 2018), this was regularised using the *Extended Bayesian Information Criterion Graphical LASSO* during network estimation. This approach attempts to limit identification of spurious edges, or connections, between nodes producing a more parsimonious model (Epskamp and Fried, 2018).

Centrality indices were computed to suggest the symptom node with the greatest influence, that is, connectedness, within the network as those with the greatest values more likely to drive change in other indicators (Robinaugh et al., 2016; Epskamp et al., 2018). Four centrality indices were imputed: *Strength*, indicating direct connectedness in the network; *Closeness*, indirect connectedness to other nodes in the network; *Betweenness*, presence on influential paths between other nodes; and *Expected Influence*, the cumulative strength and influence of a node in the network penalised for negative connections (Robinaugh et al., 2016; Epskamp et al., 2018). This metric integrates positive and negative associations between nodes to provide a summative indicator of a nodes importance in the network, in this case, the influence of an individual symptom on the wider network (Robinaugh et al., 2016). *Expected Influence* has been regarded as most valuable in understanding the presentation and clinical importance of symptoms in disorders (see Robinaugh et al., 2016; Spiller et al., 2020), and thus is considered as the primary indicator of node influence in this study.

### Network comparison test

The Network Comparison Test (NCT) assesses structural and centrality differences between group networks. It determines if two networks significantly differ by randomly reshuffling data to produce test statistics (van Borkulo et al., 2022).

The test pools and resamples data from each group, mitigating issues from comparing unequal groups. It checks for network invariance, testing if connections between networks are similar, and global strength invariance, assessing if nodes are equally connected across networks (van Borkulo et al., 2022). To ensure accuracy, the comparison between the three sub-samples drawn from each nation was tested with 1,000 iterations (van Borkulo et al., 2022) in each of three comparisons: *Ghana and Kenya*, *Ghana and Nigeria*, *Kenya and Nigeria.* The code for these analyses is provided in Supplementary File 1.

## Results

### Bereaved sample

The study analysed data from 1,554 respondents (61.57% of the total) from three African countries who experienced bereavement throughout life. The sample was gender-balanced (50.71% male), mostly in committed relationships (55.34%), employed (62.42%) and university-educated (91.63%). Country-specific demographics are in Table 2.

**Table 2.** Demographic overview for total bereaved sample, and stratified by country sub-sample

	Overall, *N* = 1,554	Ghana, *n* = 290	Kenya, *n* = 619	Nigeria, *n* = 645	Test statistic	*p*-value^a^
Sex^b^					0.4617	0.8
Male	788 (50.71%)	151 (52.07%)	308 (49.76%)	329 (51.01%)		
Female	766 (49.29%)	139 (47.93%)	311 (50.24%)	316 (48.99%)		
Age^a^	31.33 (9.17)	29.64 (8.37)	30.90 (9.07)	32.49 (9.46)	26.1475	<0.001
Relationship status^b^					5.8687	0.053
In a committed relationship/married	860 (55.34%)	142 (48.97%)	351 (56.70%)	367 (56.90%)		
Not in a committed relationship/not married	694 (44.66%)	148 (51.03%)	268 (43.30%)	278 (43.10%)		
Employment status^a^					10.6590	0.2
Not in employment, seeking work	472 (30.37%)	87 (30.00%)	189 (30.53%)	196 (30.39%)		
Not in employment, not seeking work	112 (7.21%)	25 (8.62%)	34 (5.49%)	53 (8.22%)		
Full–time employed	591 (38.03%)	108 (37.24%)	238 (38.45%)	245 (37.98%)		
Part–time employed	287 (18.47%)	48 (16.55%)	129 (20.84%)	110 (17.05%)		
Voluntary work	92 (5.92%)	22 (7.59%)	29 (4.68%)	41 (6.36%)		
Highest educational attainment^b^					13.2705	0.039
Never been through formal education	1 (0.06%)	0 (0.00%)	0 (0.00%)	1 (0.16%)		
Primary school	2 (0.13%)	1 (0.34%)	1 (0.16%)	0 (0.00%)		
Secondary school	127 (8.17%)	35 (12.07%)	53 (8.56%)	39 (6.05%)		
College/university	1,424 (91.63%)	254 (87.59%)	565 (91.28%)	605 (93.80%)		
Experienced a bereavement in past year^b^	1,088 (70.01%)	221 (76.21%)	412 (66.56%)	455 (70.54%)	8.9029	0.012
Number of bereavements in previous 5 years^a^	3.18 (1.99)	3.23 (2.00)	3.20 (2.01)	3.14 (1.97)	1.0605	0.6
Probable PGD^b^	195 (12.55%)	35 (12.07%)	92 (14.86%)	68 (10.54%)	5.4465	0.066
Total PGD score^a^	16.56 (6.68)	15.69 (6.53)	17.26 (6.95)	16.29 (6.43)	11.6988	0.003

a Kruskal-Wallis rank sum test.

b Pearson’s Chi-squared test; ^2^Kruskal-Wallis rank sum test.

All had experienced the loss of a loved one in their lifetime, with the majority reporting exposure to at least one bereavement in the previous year (70.01%). Participants reported on average 3.18 (SD = 1.99) bereavements in the previous 5 years. When queried which was the most significant bereavement participants had experienced in the past, most reported the death of a parent (*n* = 635, 40.86%). Further details of bereavement endorsement are provided in Supplementary File 1.

### Country sub-samples

Minor demographic differences were noted between groups in the sample stratified by country of origin (see Table 2). The Nigerian sample was found to be significantly older, and reported higher educational attainment, relative to the other country sub-samples. Pairwise comparisons between samples are provided in Supplementary File 1.

**Figure 1.**
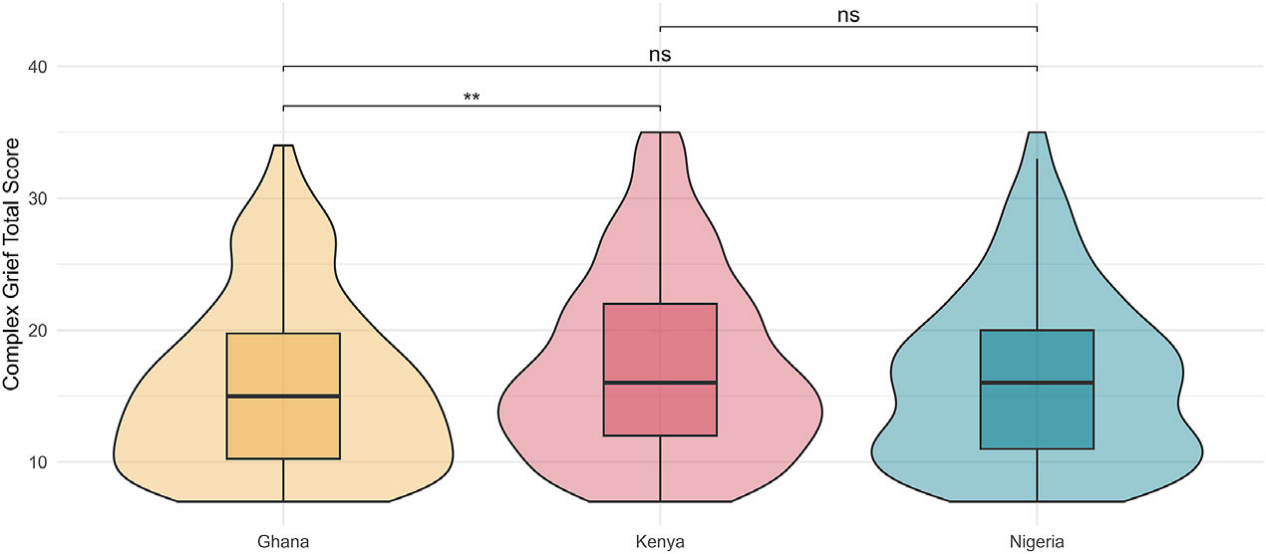
Comparison of PGD total scores between sub-sample groups. Note: Data distribution and frequency are represented by the probability density plot, and summative statistics represented by boxplot internally; depicting the median (central marker), interquartile range (box) and total data range (whiskers). *p < .05, **p < .01, ***p < .001, ns = non-significant.

No statistically significant difference was observed comparing those who would screen positively for PGD (*χ* ^2^ = 5.4465, *p* = .06), however, a significant difference was observed between groups on total ICGR score (*H* (2) = 11.6988, *p* < .01). The distribution of these data and results of Wilcoxon test comparisons are shown via violin plot in Figure 1. Those surveyed from Ghana had a lower mean score on the ICGR compared to the Kenya samples (Wilcoxon W = 77,806, *p* < .01). The comparisons between the Ghana-Nigeria and Kenya-Nigeria samples were not statistically significant.

A statistically significant difference was noted regarding the experience of past year bereavement (*χ* ^2^ = 8.9029, *p* < .05), with the Kenyan sample less likely to report experience of bereavement in the previous year relative to the Ghana sample. The Nigerian sample reported greater educational attainment relative to the Ghana sample. Full details of these post-hoc comparisons are available in Supplementary File 1.

## Network analysis and comparison

**Figure 2.**
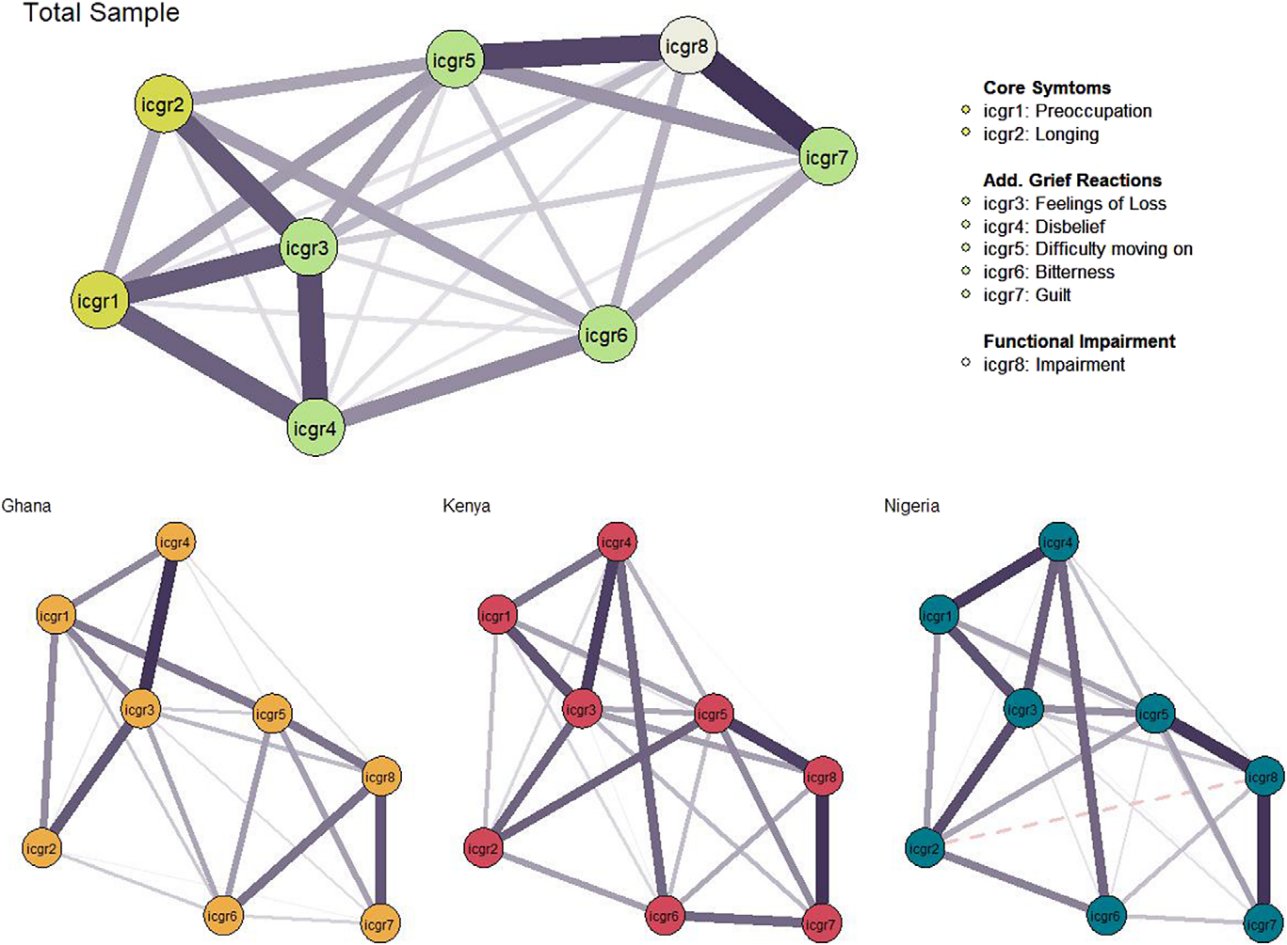
Network structures for total prolonged grief disorder network, and sub-sample networks. Note: Nodes represent individual items of the Inventory of Complicated Grief Revised, connecting lines represent edge weights with greater thickness, indicating greater strength of association. Negative associations are represented by a dashed line. Layout based on the averaged spring algorithm across networks; placing the nodes with greater connectedness closer proximally, and plotting node with the greatest expected influence more centrally.

Network structures were estimated for the total bereaved sample, in addition to each of the three country sub-samples (see Figure 2). Extensive connectedness was found in the total sample network containing 26 of 28 nonzero edges (92.86%), and each of the sub-sample networks likewise containing majority of nonzero edges (Ghana, 92.31%; Nigeria, 92.31%; Kenya, 85.71%), suggesting ICGR grief indicators to be strongly and positively correlated. Visual inspection suggested a relatively stable network structure with *Feelings of Loss* (ICGR3) as an influential node, strongly connected to others in the network. *Preoccupation* with the deceased (ICGR5) and *feelings of guilt* (ICGR7) were most connected to functional impairment in the total sample network. The overall stability of the network was favourable with correlation stability ranging from. 361 (Betweenness) to. 950 (Strength; Expected Influence). Details of network stability tests are available in Supplementary File 1.

**Figure 3.**
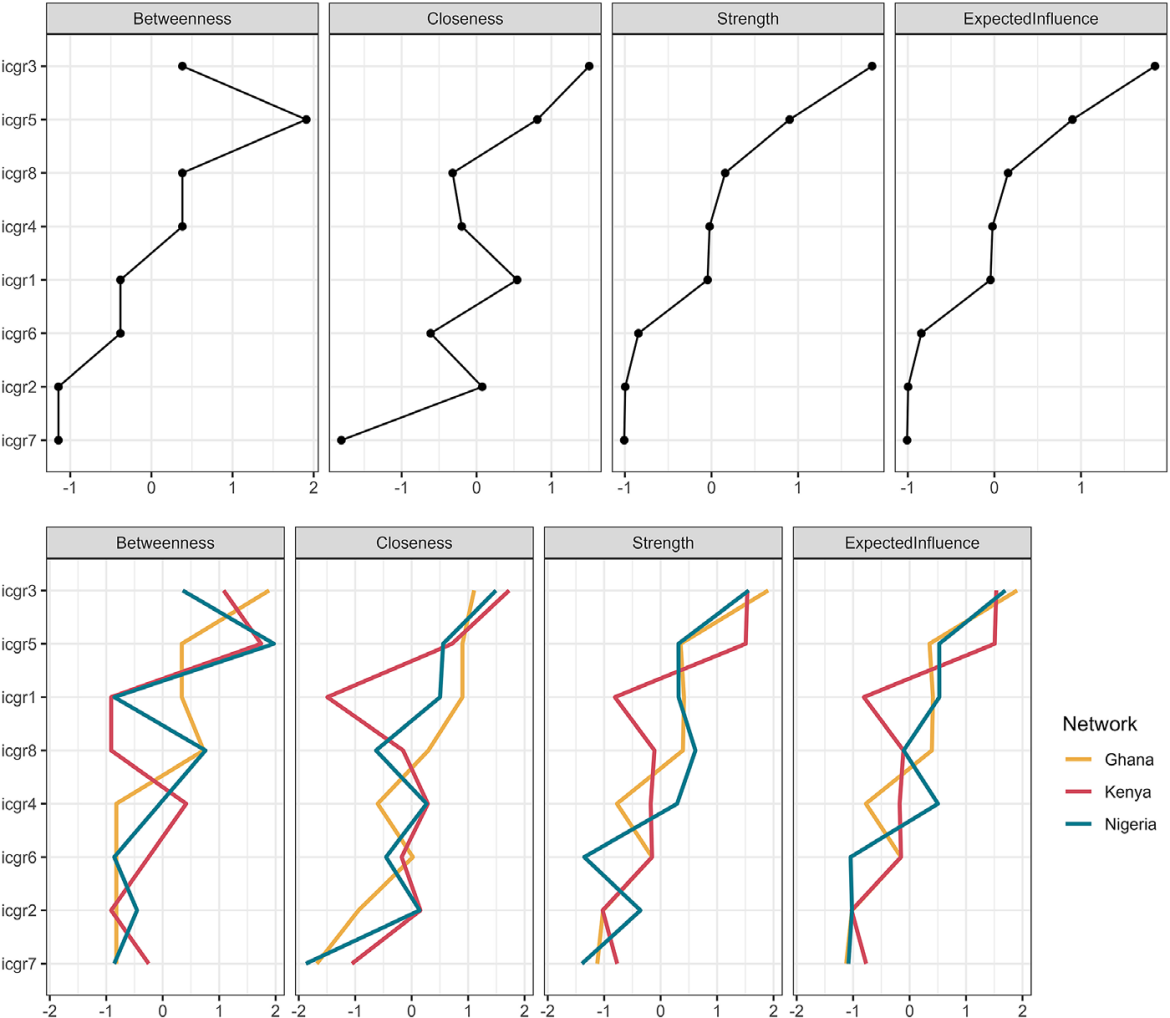
Node centrality indices for total sample network and country stratified samples. Note: Values on the *x*-axis represent standardised scores indicating centrality indices for each ICGR item. Centrality indices for the total sample network are plotted in panel A, and for each country sub-sample network (Ghana = Yellow, Kenya = Red, Nigeria = Green) in panel B.

Similar connectivity was observed among nodes in the three sub-sample networks (see Figure 2). Node arrangement was regularised by average layout between these networks to aid in presentation and interpretation. The most notable difference between networks was the connectivity between *Feelings of Longing* (ICGR2), *Feelings of Loss* (ICGR3), and *Disbelief* (ICGR4); with these nodes appearing more strongly connected in the Ghana network. *Feelings of Loss* (ICGR3) and *Disbelief* (ICGR4) were more strongly connected in the Kenya network. Consistent with expectations, node Expected Influence for the sub-sample networks was broadly reminiscent of the total sample indices with some variations (see Figure 3). Differences were observed across Expected Influence Across sub-samples *Feelings of Loss* (ICGR3) was the most central node in the total sample network, and in the Ghana and Nigeria sample networks. Notably, in the Kenyan sample, relative to the other sub-samples: *Difficulty moving* on (ICGR5) displayed greater expected influence and was comparable to *Feelings of Loss* (ICGR3) (see Figure 3).

Sub-sample networks were statistically compared using the NCT function highlighting *network and global strength invariance* (see Supplementary File 1). Global strength metrics were similar for all sub-sample networks (Ghana S = 3.455, Kenya S = 3.573, Nigeria S = 3.598), and no statistically significant differences were found between networks (see Supplementary File 1). There was a statistically significant difference in network structure between the Ghana-Kenya networks (*M* = 0.2247, *p* < .05), and Kenya-Nigeria networks (*M* = 0.2285, *p* < .05) network comparisons.

Statistically significant edge-weight differences were inspected between networks (see Supplementary File 1). In both network comparisons, the edge strength between “*Bitterness*” and “*Guilt*” (ICGR6—ICGR7) was greater in the Kenya sample network relative to the Nigeria sample (Δ*r* = 0.1191, *p* < .05) and Ghana sample (Δ*r* = 0.1406, *p* < .05). Two further significant differences were identified in individual edge-weights between the Kenya and Ghana networks with both being more strongly connected in the Kenya sample network; between “*Longing*” and “*Difficulty moving on*” (ICGR2—ICGR5; Δ*r* = 0.2247, *p* < .01), and between “*Disbelief*” and “*Guilt*” (ICGR4—ICGR6; Δ*r* = 0.2247, *p* < .01).

## Discussion

This study aimed to enhance understanding of ICD-11 PGD in an African sample through network analysis, examining the disorder’s characteristics. Hypothesis 1, that indicators of *emotional pain* and *feelings of loss* would present as the most central in the network, was partially supported. While indicators of emotional pain captured by the ICGR “additional grief reactions” were influential, preoccupation and longing had a low impact, with longing’s influence notably low despite being a hallmark of the diagnostic concept in ICD-11 criteria, and being the most often endorsed item (*n* = 659, 26%). This suggests that while longing is common in grief, it may not alone indicate a pathological response (Robinaugh et al., 2014). The study highlights symptoms with high expected influence in network models, which may be key targets for intervention (Robinaugh et al., 2016).

*Feelings of Loss* (ICGR3), that is, profound sense of loss involving feeling a part of one’s self is lost, was consistently found to exhibit the highest strength centrality in these networks. In the Kenya sample, however, both *Feelings of Loss* (ICGR3) and *Difficulty moving on* (ICGR5) were equally most central. These results align with previous studies that relied on Western countries samples, prior to ICD-11 PGD definitions and standardised measures suggesting indicators of *emotional pain* to be central to disordered grief (Robinaugh et al., 2014, 2016). While previous studies have modelled this as an independent node, the current study, conceptualised potential indicators of emotional pain through the additional grief reactions contained in the ICGR. The centrality of these more specific difficulties is argued to indicate these to be drivers of distress in the disordered grief network, that is, those profound feelings of loss related to bereavement may act as triggers maintaining other recognised PGD symptoms in these contexts. The importance of these indicators of emotional pain indicated by these results, coded as ‘additional grief reactions’ in the ICGR, should, therefore, be considered.

The results of the current study specifically highlight *Feelings of Loss* (ICGR3) and *Difficulty moving on* (ICGR5) as potential key targets for intervention. They reflect the connection between a loss of self and a loss of ability to move on with one’s life. It is worth noting that these items on the ICGR, while not developed to explicitly relate to the constructs of *Preoccupation* and *Longing* defined as core to diagnosis by ICD-11 criteria, may be considered conceptually similar to these based on face validity. It may also be noted that the “*Feelings of Loss*” node may be argued to hold face validity for the concept of *identity disruption*, core to DSM-5-TR criteria for PGD (Eisma, 2023). A profound feeling of loss, including losing part of one’s self may be considered as a central marker of PGD-related distress and potential target for intervention across diagnostic frameworks.

Moreover, as PGD is effectively conceptualised as a unidimensional construct and does not warrant faceting symptoms into sub domains/clusters (see Maciejewski et al., 2016; Schakowski et al., 2023), researchers and clinicians should remain mindful of variations in individual-level symptom associations as exampled through network analyses in the current and previous works (e.g., Maccallum et al., 2017; Malgaroli et al., 2018) and how these align with ICD-11 criteria.

Hypothesis 2, that symptom networks would remain stable across country sub-samples, was partially supported; visual analysis of the PGD network across three sub-samples showed similarities, with tests confirming no significant differences in global network strength. However, Network Invariance differed notably between the Kenya sample and those from Nigeria and Ghana. In Figure 2, Feelings of Loss (ICGR3) appeared central in all samples, but in Kenya, difficulty moving on (ICGR5) also emerged as a key symptom. Additionally, the Kenya sample showed less centrality and influence for preoccupation compared to the others. As this study design is cross-sectional, high centrality indicates stronger associations with adjacent symptoms that do not ascribe causality in influence, that is, these may be bidirectional relationships (Borsboom et al., 2021). Central symptoms can be useful targets for intervention and prioritising symptoms of “*Feelings of Loss*” and “*Difficulty moving on*” for treatment may have a positive impact on all other symptoms as they are more closely associated. For instance, based on these findings interventions may prioritise reducing feelings of loss, which lead to grief acceptance and moving on, ultimately reducing yearning and ultimately deactivating the network of distress associated with PGD difficulties.

A notable exception to the first study hypothesis and finding from the total sample network, of the influence of *preoccupation* in the PGD network, was found in this comparison and metrics from the Kenya network. Results from this sub-sample uniquely showed that *preoccupation* exhibited markedly lower expected influence relative to the other samples. Prior evidence has highlighted alternate trajectories and expressions of disordered grief between 3 and 25 months post-bereavement, suggesting that difficulties characteristic of ICD-11 PGD effectively capture change over time (Bonanno and Malgaroli, 2020). Results of the current study distinguishing patterns of disordered grief associated with the Kenya sample may, therefore, be attributed in part to the greater reporting of past-year bereavement in this group. Further investigation is warranted to examine the network expression of disordered grief longitudinally in these contexts to better understand the effects of time since bereavement in changing symptom-level associations.

Additionally noted is the relative greater connectedness of individual edges exhibited by the Kenya network. These results highlighted the cconnection between feelings of “*Guilt*” and “*Bitterness*” relative to the other samples, and feelings of “*Disbelief,*” relative to the Ghana network. Taken together, it is suggested that grief-related guilt may be of particular concern in certain contexts, and that the risk for disordered grief reactions should be considered in light of group norms.

Cultural differences in grief processes should, therefore, be considered in PGD assessment. For instance, in Kenya among some ethnic groups, such as the Luo people, grieving rituals are of great importance and multifaceted, requiring collective and extended focus on the dead and the bereaved (Shino, 1997). For this reason, *preoccupation* with the deceased may not be considered pathological and related to other indicators of PGD in this context. It should, however, be noted that there are diverse cultural and religious influences to grief practices across African contexts with great variation in typical aspects of communal and individual mourning (Njue et al., 2015). Further research is hence needed to assess standardised measurement of these indicators of grief disorders; to recognise how these are understood in different cultural contexts, and to the value of using available culturally adapted instruments.

Killikelly et al. (2020) presented the development of additional items for the International Prolonged Grief Disorder Scale exampling this, a purpose-built measure of ICD-11 PGD, presented as a “Cultural Supplement” containing additional items validated with German and Chinese speaking respondents. The initial validation of this addendum presented an argument for the use of a standardised measure across cultures, and additional culture-specific items to provide better-informed assessment and clinical decision making (Killikelly et al., 2020). Paired with the findings of the current study, it may be recommended that supplement items be similarly developed in African community contexts to ensure culturally relevant and accurate measurement.

Correctly identifying PGD and distinguishing it from other stressor-related disorders is crucial for understanding distress and devising effective treatments (Maercker and Eberle, 2022). Cognitive therapies, effective for mood and stress disorders, must be tailored for PGD, focusing on loss and moving on to reduce its persistence (Smith and Ehlers, 2021). Clinicians should target these key symptoms and their connections to disrupt distress networks (Borsboom et al., 2021). For example, therapies addressing loss and rumination could lessen central distress nodes, and integrating self-compassion might help reduce guilt-related functional impairment in PGD (Alonso-Llácer et al., 2020).

A recent review and meta-analytic evidence have indicated that targeted intervention delivered at least 6 months post-bereavement, and assessed using instruments aligned to ICD-11 symptom criteria, such as the ICGR, may be most effective in treating and evaluating disordered grief (Johannsen et al., 2019, 2022). The need for individualised assessment and effective treatment of disordered grief are highlighted.

Further noted is the potential to qualify a “moderate” and “strict” presentation of PGD. These pathological grief responses are similarly characterised by functional impairment, and delineated by the severity/chronicity of symptom presentation with “*moderate PGD*” defined by symptoms appearing “sometimes,” “often,” or “always”; and “*strict PGD*” limited only to those symptom presentations endorsed as “frequently” and “always” (Shevlin et al., 2023). The current study adopted an alternative, dimensional, approach to PGD assessment; however, future research is warranted examining the network structure of PGD symptomology carried by symptom severity in line with these developmental diagnostic algorithms to better understand dimensionality in these differing presentations.

## Strength & Limitations

This study offers a significant insight into Prolonged Grief Disorder (PGD) using a non-Western, gender-balanced sample, highlighting PGD’s global relevance and its impact on previously underrepresented groups in grief research (Hilberdink et al., 2023; Maccallum et al., 2023).

However, its cross-sectional nature and lack of detailed assessment of time since bereavement limits the ability to infer causality, and the use of proxy measurement, while effectively used to screen and evidence PGD-related difficulties (see Killikelly and Maercker, 2023), may affect results. Future research should include longitudinal studies with diverse mental health indicators and culturally adapted PGD measures specific to current diagnostic criteria (see Eisma, 2023). Nevertheless, the potential for these results to be influenced by the measurement tool applied should be acknowledged. The findings, derived from a well-educated, online panel, should be generalised cautiously. The study also focuses solely on PGD symptoms, overlooking potential associations with other psychopathologies like PTSD and depression. Conducted in English-speaking countries, the research may not fully represent non-English speakers, underscoring the need for studies in multiple languages to validate the findings across different cultural contexts. Including specific cultural adaptation, for instance, the International Prolonged Grief Disorder Scale (Killikelly et al., 2021) and cultural supplement (Killikelly and Maercker, 2023), developed to comprehensively measure specific ICD-11 PGD symptoms across diverse cultural contexts. Future work should also explore PGD in treatment-seeking populations and include clinician assessments to better capture the disorder’s clinical significance.

While those sampled were matched to demographic distribution (aged, sex) based on their respective countries census data, they may possess other shared demographic characteristics that may not be representative of the wider population (Ben-Ezra et al., 2020). Likewise, the current study sample was limited to those who reported experiencing bereavement throughout their life and applied algorithmic scoring of ICGR responses to assess probable PGD, an established practice (see Bagcaz and Kilic, 2023). The results of the current study should be interpreted and generalised beyond the current study cautiously for these reasons.

Assessment of network symptomology was also limited to these indicators of PGD. It should be noted that other psychopathologies may be associated with disordered grief, such as PTSD and depression (Bonanno et al., 2007). Future research may consider further exploring relationships between PGD and related symptomology (see Maccallum et al., 2017).

Data were collected in English across countries where English is official, yet minority languages were not included, potentially limiting cross-cultural applicability. Standardised measurements facilitate comparison but might not fully capture non-English speakers’ experiences, underscoring the need for validation across languages as per the ICD-11 Task Force goals (Maercker et al., 2013; Maercker and Eberle, 2022; Eisma, 2023). Future studies should extend to treatment-seeking individuals and those at higher PGD risk, incorporating clinical assessments to validate screening tools.

The network analysis underscores the significance of loss in PGD and suggests that cultural considerations are crucial in assessment and diagnosis. While the findings offer potential intervention targets in PGD, especially in African contexts, further research is needed to confirm their clinical impact.

## Supporting information

Robinson et al. supplementary material Robinson et al. supplementary material

## Data Availability

Data corresponding to these analyses are not made publicly available to protect the identity of participants. These data may be made available from the corresponding author on reasonable request.

## Supplementary material

The supplementary material for this article can be found at http://doi.org/10.1017/gmh.2024.54.
