# Supplementary material for: Prolonged grief disorder symptomology in three African countries: A network analysis and comparison
Source: Glob Ment Health (Camb). 2024 Apr 29;11:e57. doi: 10.1017/gmh.2024.54 (PMC11094551; doi:10.1017/gmh.2024.54)
Supplement: Robinson et al. supplementary material [file S2054425124000542sup001.zip › S2054425124000542sup001.html]

R Markdown: Prolonged Grief Disorder Symptomology in Three African Countries, A Network Analysis and Comparison


# R Markdown: Prolonged Grief Disorder Symptomology in Three African Countries, A Network Analysis and Comparison

#### M Robinson

#### 18 Jul 2023

### Setup and Files

```
knitr::opts_chunk$set(echo = TRUE, warning = FALSE, message = FALSE)
options(width = 200)
options(scipen = 999, digits = 4)                                               # Remove scientific notation and set sig figures for tables.

#  Packages for Analysis
library("bootnet")
```

```
## Loading required package: ggplot2
```

```
## Warning: package 'ggplot2' was built under R version 4.2.2
```

```
## This is bootnet 1.5
```

```
## For questions and issues, please see github.com/SachaEpskamp/bootnet.
```

```
library("networktools")
library("NetworkComparisonTest")
library("qgraph")
```

```
## Warning: package 'qgraph' was built under R version 4.2.2
```

```
library("rstatix")
```

```
## Warning: package 'rstatix' was built under R version 4.2.2
```

```
## 
## Attaching package: 'rstatix'
```

```
## The following object is masked from 'package:stats':
## 
##     filter
```

```
#  Packages for Presentation
library("flextable")
```

```
## Warning: package 'flextable' was built under R version 4.2.3
```

```
library("gtsummary")
```

```
## Warning: package 'gtsummary' was built under R version 4.2.2
```

```
## #BlackLivesMatter
```

```
## 
## Attaching package: 'gtsummary'
```

```
## The following objects are masked from 'package:flextable':
## 
##     as_flextable, continuous_summary
```

```
library("tidyverse")
```

```
## Warning: package 'tidyverse' was built under R version 4.2.2
```

```
## Registered S3 method overwritten by 'modelr':
##   method         from
##   print.resample mgm
```

```
## ── Attaching packages ─────────────────────────────────────────────────────────────────────────────────────────────────────────────────────────────────────────────────────────────── tidyverse 1.3.2 ──
## ✔ tibble  3.1.8      ✔ dplyr   1.0.10
## ✔ tidyr   1.2.1      ✔ stringr 1.5.0 
## ✔ readr   2.1.3      ✔ forcats 0.5.2 
## ✔ purrr   1.0.1
```

```
## Warning: package 'tidyr' was built under R version 4.2.2
```

```
## Warning: package 'readr' was built under R version 4.2.2
```

```
## Warning: package 'purrr' was built under R version 4.2.2
```

```
## Warning: package 'dplyr' was built under R version 4.2.2
```

```
## Warning: package 'stringr' was built under R version 4.2.2
```

```
## ── Conflicts ────────────────────────────────────────────────────────────────────────────────────────────────────────────────────────────────────────────────────────────────── tidyverse_conflicts() ──
## ✖ purrr::compose() masks flextable::compose()
## ✖ dplyr::filter()  masks rstatix::filter(), stats::filter()
## ✖ dplyr::lag()     masks stats::lag()
```

```
#  Set Working Directory and R Environment Items
setwd("D:/zb. Publication Records/ha. Prolonged Grief Disorder/")
data <- read.csv("DSAS_Master_CSV.csv")
data[data=="-99"]<-NA
PGD <- data |> filter(adnme6 == 1) |> dplyr::select("icgr1":"icgr8")
ltm::cronbach.alpha(PGD)
```

```
## 
## Cronbach's alpha for the 'PGD' data-set
## 
## Items: 8
## Sample units: 1554
## alpha: 0.903
```

```
GhanaNet   <- data |> filter(adnme6 == 1) |> filter(Country == 1) |> dplyr::select("icgr1":"icgr8")
KenyaNet   <- data |> filter(adnme6 == 1) |> filter(Country == 2) |> dplyr::select("icgr1":"icgr8")
NigeriaNet <- data |> filter(adnme6 == 1) |> filter(Country == 3) |> dplyr::select("icgr1":"icgr8")
ltm::cronbach.alpha(GhanaNet)
```

```
## 
## Cronbach's alpha for the 'GhanaNet' data-set
## 
## Items: 8
## Sample units: 290
## alpha: 0.901
```

```
ltm::cronbach.alpha(KenyaNet)
```

```
## 
## Cronbach's alpha for the 'KenyaNet' data-set
## 
## Items: 8
## Sample units: 619
## alpha: 0.919
```

```
ltm::cronbach.alpha(NigeriaNet)
```

```
## 
## Cronbach's alpha for the 'NigeriaNet' data-set
## 
## Items: 8
## Sample units: 645
## alpha: 0.888
```

```
subscales <- list("Core Symtoms"           =c(1:2), 
                  "Add. Grief Reactions"   =c(3:7),
                  "Functional Impairment"  =c(8))

labels    <-    c("Preoccupation", "Longing", 
                  "Loss", "Disbelief","Difficulty moving on", "Bitterness", "Guilt", 
                  "Impariment")
```

## R Markdown

## Descriptives

```
labelled_data <- jmvReadWrite::read_omv("D:/zb. Publication Records/ha. Prolonged Grief Disorder/Africa DSAS_Master.omv")

PGD_diag_data <- data |>                                                        # Screening for PGD according to ICD-11 Criteria
  mutate(icgr1_case = case_when(icgr1 >= 4 ~ 1, icgr1 < 4 ~ 0),                 # PGD items defined as present with repose of 4 | 5
         icgr2_case = case_when(icgr2 >= 4 ~ 1, icgr2 < 4 ~ 0),                 # to any ICGR item (Ben-Ezra et al., 2020).
         icgr3_case = case_when(icgr3 >= 4 ~ 1, icgr3 < 4 ~ 0),
         icgr4_case = case_when(icgr4 >= 4 ~ 1, icgr4 < 4 ~ 0),
         icgr5_case = case_when(icgr5 >= 4 ~ 1, icgr5 < 4 ~ 0),
         icgr6_case = case_when(icgr6 >= 4 ~ 1, icgr6 < 4 ~ 0),
         icgr7_case = case_when(icgr7 >= 4 ~ 1, icgr7 < 4 ~ 0),
         icgr8_case = case_when(icgr8 >= 4 ~ 1, icgr8 < 4 ~ 0)) |> 
  mutate(PGD_Crti1 = case_when(icgr1 >= 4 ~ 1,                                  # Should endorse longing [icgr1] or preoccupation [icgr2].
                               icgr1 < 4 ~ 0,
                               icgr2 >= 4 ~ 1,
                               icgr2 < 4 ~ 0),
         PDG_Crit2 = case_when(icgr3_case + icgr4_case + icgr5_case +           # Should endorse three or more add. grief symptoms.
                               icgr6_case + icgr7_case >= 3 ~ 1,
                               icgr3_case + icgr4_case + icgr5_case + 
                               icgr6_case + icgr7_case < 3 ~ 0),
         PDG_Crit3 = case_when(icgr8 >= 4 ~ 1,                                  # Should endorse impairment [icgr8].
                               icgr8 < 4 ~ 0),
         PDG_diag  = case_when(PGD_Crti1 + PDG_Crit2 + PDG_Crit3 == 3 ~ 1,      # If all criteria are met code 1 "Disorder Present",
                               PGD_Crti1 + PDG_Crit2 + PDG_Crit3 < 3 ~ 0))  |>  # Otherwise code 0 "Disorder not present".
    rowwise() |>                                                                # Await confirmation from Yafit.
    mutate(ICGR_tot = sum(icgr1+icgr2+icgr3+icgr4+icgr5+icgr6+icgr7)) |> 
    ungroup()


PGD_diag_data |> 
  dplyr::select(icgr1_case:PDG_diag) |> 
  tbl_summary()
```

| **Characteristic** | **N = 2,524**1 |
| --- | --- |
| icgr1\_case | 418 (17%) |
| icgr2\_case | 659 (26%) |
| icgr3\_case | 425 (17%) |
| icgr4\_case | 526 (21%) |
| icgr5\_case | 211 (8.4%) |
| icgr6\_case | 569 (23%) |
| icgr7\_case | 209 (8.3%) |
| icgr8\_case | 155 (6.1%) |
| PGD\_Crti1 | 418 (17%) |
| PDG\_Crit2 | 294 (12%) |
| PDG\_Crit3 | 155 (6.1%) |
| PDG\_diag | 98 (3.9%) |
|  |  |
| --- | --- |
| 1 n (%) | |

```
## Total Dataset Summary Table
labelled_data |> 
  dplyr::select("Country", "Sex", "Age", "Marit", "Employ", "Educ", "adnme6") |> 
  tbl_summary(by = "Country", 
              label = list(
                Marit  ~ "Marital Status",
                Employ ~ "Employment Status",
                Educ   ~ "Highest Educational Attainment",
                adnme6 ~ "Experienced Loss"),
              type = list(
                Age    ~ "continuous"),
              digits = list(all_continuous() ~ 2, all_categorical() ~ c(0, 2)),  
              missing = "no") |> 
  add_p(list(all_categorical() ~ "chisq.test",
             all_continuous() ~ "kruskal.test"),  include = everything()) |> 
    modify_header(statistic ~ "**Test Statistic**") |> 
    modify_caption("**Demographic Characteristics by Country (Total Sample)**") |> 
  add_overall()
```

**Demographic Characteristics by Country (Total Sample)**

| **Characteristic** | **Overall**, N = 2,5241 | **Ghana**, N = 5001 | **Kenya**, N = 1,0061 | **Nigeria**, N = 1,0181 | **Test Statistic** | **p-value**2 |
| --- | --- | --- | --- | --- | --- | --- |
| Sex |  |  |  |  | 0.1424 | >0.9 |
| Male | 1,273 (50.44%) | 250 (50.00%) | 505 (50.20%) | 518 (50.88%) |  |  |
| Female | 1,251 (49.56%) | 250 (50.00%) | 501 (49.80%) | 500 (49.12%) |  |  |
| Age | 28.00 (25.00, 35.00) | 27.00 (24.00, 32.00) | 27.50 (24.00, 33.75) | 30.00 (25.00, 37.00) | 62.8117 | <0.001 |
| Marital Status |  |  |  |  | 15.0188 | <0.001 |
| In a committed relationship/Married | 1,346 (53.33%) | 228 (45.60%) | 553 (54.97%) | 565 (55.50%) |  |  |
| Not in a committed relationship/ Not married | 1,178 (46.67%) | 272 (54.40%) | 453 (45.03%) | 453 (44.50%) |  |  |
| Employment Status |  |  |  |  | 9.5402 | 0.3 |
| Not in employment, seeking work | 774 (30.67%) | 157 (31.40%) | 318 (31.61%) | 299 (29.37%) |  |  |
| Not in employment, not seeking work | 184 (7.29%) | 41 (8.20%) | 65 (6.46%) | 78 (7.66%) |  |  |
| Full-time employed | 937 (37.12%) | 176 (35.20%) | 369 (36.68%) | 392 (38.51%) |  |  |
| Part-time employed | 465 (18.42%) | 84 (16.80%) | 198 (19.68%) | 183 (17.98%) |  |  |
| Voluntary work | 164 (6.50%) | 42 (8.40%) | 56 (5.57%) | 66 (6.48%) |  |  |
| Highest Educational Attainment |  |  |  |  | 24.4867 | <0.001 |
| Never been through formal education | 1 (0.04%) | 0 (0.00%) | 0 (0.00%) | 1 (0.10%) |  |  |
| Primary School | 5 (0.20%) | 4 (0.80%) | 1 (0.10%) | 0 (0.00%) |  |  |
| Secondary School | 198 (7.84%) | 54 (10.80%) | 83 (8.25%) | 61 (5.99%) |  |  |
| College / University | 2,320 (91.92%) | 442 (88.40%) | 922 (91.65%) | 956 (93.91%) |  |  |
| Experienced Loss | 1,554 (61.57%) | 290 (58.00%) | 619 (61.53%) | 645 (63.36%) | 4.0716 | 0.13 |
|  |  |  |  |  |  |  |
| --- | --- | --- | --- | --- | --- | --- |
| 1 n (%); Median (IQR) | | | | | | |
| 2 Pearson's Chi-squared test; Kruskal-Wallis rank sum test | | | | | | |

```
## Bereaved Sample Summary Table; Table 1
labelled_data |> 
  filter(adnme6 == "Yes") |> 
  mutate(lost5y = as.numeric(lost5y)) |> 
  dplyr::select("Country", "Sex", "Age", "Marit", "Employ", "Educ", "lost1y", "lost5y", "lostperson", "ICGR", "Prob_PGD", "ICGR_tot") |> 
  tbl_summary(by = "Country", 
              label = list(
                Marit  ~ "Marital Status",
                Employ ~ "Employment Status",
                Educ   ~ "Highest Educational Attainment",
                lost1y ~ "Experienced a Bereavement in Past Year",
                lost5y ~ "Number of Bereavements in Previous 5 Years",
                lostperson ~ "Most Significant Bereavement",
                ICGR   ~ "Complex Grief Criteria Met",
                Prob_PGD ~ "Probable PGD (Martin Calculation)",
                ICGR_tot ~ "Total PGD Score"),
              type = list(
                Age    ~ "continuous",
                lost5y ~ "continuous",
                ICGR_tot ~ "continuous"),
              statistic = all_continuous() ~ "{mean} ({sd})",
              digits = list(all_continuous() ~ 2, all_categorical() ~ c(0, 2)),  
              missing = "no") |> 
  add_p(list(all_categorical() ~ "chisq.test",
             all_continuous()  ~ "kruskal.test"),  include = everything()) |> 
    modify_header(statistic ~ "**Test Statistic**") |> 
    modify_caption("**Demographic Characteristics by Country (Bereaved Sample)**") |> 
  add_overall() # |> as_flex_table() |> save_as_docx(path = "Table1.docx")
```

**Demographic Characteristics by Country (Bereaved Sample)**

| **Characteristic** | **Overall**, N = 1,5541 | **Ghana**, N = 2901 | **Kenya**, N = 6191 | **Nigeria**, N = 6451 | **Test Statistic** | **p-value**2 |
| --- | --- | --- | --- | --- | --- | --- |
| Sex |  |  |  |  | 0.4617 | 0.8 |
| Male | 788 (50.71%) | 151 (52.07%) | 308 (49.76%) | 329 (51.01%) |  |  |
| Female | 766 (49.29%) | 139 (47.93%) | 311 (50.24%) | 316 (48.99%) |  |  |
| Age | 31.33 (9.17) | 29.64 (8.37) | 30.90 (9.07) | 32.49 (9.46) | 26.1475 | <0.001 |
| Marital Status |  |  |  |  | 5.8687 | 0.053 |
| In a committed relationship/Married | 860 (55.34%) | 142 (48.97%) | 351 (56.70%) | 367 (56.90%) |  |  |
| Not in a committed relationship/ Not married | 694 (44.66%) | 148 (51.03%) | 268 (43.30%) | 278 (43.10%) |  |  |
| Employment Status |  |  |  |  | 10.6590 | 0.2 |
| Not in employment, seeking work | 472 (30.37%) | 87 (30.00%) | 189 (30.53%) | 196 (30.39%) |  |  |
| Not in employment, not seeking work | 112 (7.21%) | 25 (8.62%) | 34 (5.49%) | 53 (8.22%) |  |  |
| Full-time employed | 591 (38.03%) | 108 (37.24%) | 238 (38.45%) | 245 (37.98%) |  |  |
| Part-time employed | 287 (18.47%) | 48 (16.55%) | 129 (20.84%) | 110 (17.05%) |  |  |
| Voluntary work | 92 (5.92%) | 22 (7.59%) | 29 (4.68%) | 41 (6.36%) |  |  |
| Highest Educational Attainment |  |  |  |  | 13.2705 | 0.039 |
| Never been through formal education | 1 (0.06%) | 0 (0.00%) | 0 (0.00%) | 1 (0.16%) |  |  |
| Primary School | 2 (0.13%) | 1 (0.34%) | 1 (0.16%) | 0 (0.00%) |  |  |
| Secondary School | 127 (8.17%) | 35 (12.07%) | 53 (8.56%) | 39 (6.05%) |  |  |
| College / University | 1,424 (91.63%) | 254 (87.59%) | 565 (91.28%) | 605 (93.80%) |  |  |
| Experienced a Bereavement in Past Year | 1,088 (70.01%) | 221 (76.21%) | 412 (66.56%) | 455 (70.54%) | 8.9029 | 0.012 |
| Number of Bereavements in Previous 5 Years | 3.18 (1.99) | 3.23 (2.00) | 3.20 (2.01) | 3.14 (1.97) | 1.0605 | 0.6 |
| Most Significant Bereavement |  |  |  |  | 68.7353 | <0.001 |
| Father | 322 (20.72%) | 59 (20.34%) | 113 (18.26%) | 150 (23.26%) |  |  |
| Mother | 313 (20.14%) | 66 (22.76%) | 138 (22.29%) | 109 (16.90%) |  |  |
| Wife | 36 (2.32%) | 1 (0.34%) | 27 (4.36%) | 8 (1.24%) |  |  |
| Husband | 13 (0.84%) | 2 (0.69%) | 3 (0.48%) | 8 (1.24%) |  |  |
| Grandfather | 62 (3.99%) | 9 (3.10%) | 33 (5.33%) | 20 (3.10%) |  |  |
| Grandmother | 133 (8.56%) | 29 (10.00%) | 64 (10.34%) | 40 (6.20%) |  |  |
| Sister | 71 (4.57%) | 11 (3.79%) | 20 (3.23%) | 40 (6.20%) |  |  |
| Brother | 86 (5.53%) | 15 (5.17%) | 31 (5.01%) | 40 (6.20%) |  |  |
| Son | 22 (1.42%) | 2 (0.69%) | 14 (2.26%) | 6 (0.93%) |  |  |
| Daughter | 22 (1.42%) | 3 (1.03%) | 7 (1.13%) | 12 (1.86%) |  |  |
| Cousin | 65 (4.18%) | 7 (2.41%) | 27 (4.36%) | 31 (4.81%) |  |  |
| Niece | 14 (0.90%) | 2 (0.69%) | 7 (1.13%) | 5 (0.78%) |  |  |
| Uncle | 97 (6.24%) | 21 (7.24%) | 35 (5.65%) | 41 (6.36%) |  |  |
| Aunt | 64 (4.12%) | 12 (4.14%) | 23 (3.72%) | 29 (4.50%) |  |  |
| Grandson | 0 (0.00%) | 0 (0.00%) | 0 (0.00%) | 0 (0.00%) |  |  |
| Granddaughter | 1 (0.06%) | 0 (0.00%) | 1 (0.16%) | 0 (0.00%) |  |  |
| Other close relative | 73 (4.70%) | 16 (5.52%) | 24 (3.88%) | 33 (5.12%) |  |  |
| Close friend. | 160 (10.30%) | 35 (12.07%) | 52 (8.40%) | 73 (11.32%) |  |  |
| Complex Grief Criteria Met |  |  |  |  | 10.5238 | 0.005 |
| Criteria for Complicated Grief not met | 1,496 (96.27%) | 286 (98.62%) | 585 (94.51%) | 625 (96.90%) |  |  |
| Criteria for Complicated grief met | 58 (3.73%) | 4 (1.38%) | 34 (5.49%) | 20 (3.10%) |  |  |
| Probable PGD (Martin Calculation) | 195 (12.55%) | 35 (12.07%) | 92 (14.86%) | 68 (10.54%) | 5.4465 | 0.066 |
| Total PGD Score | 16.56 (6.68) | 15.69 (6.53) | 17.26 (6.95) | 16.29 (6.43) | 11.6988 | 0.003 |
|  |  |  |  |  |  |  |
| --- | --- | --- | --- | --- | --- | --- |
| 1 n (%); Mean (SD) | | | | | | |
| 2 Pearson's Chi-squared test; Kruskal-Wallis rank sum test | | | | | | |

```
#  Pairwise Comparisons for Sig. Differences
labelled_data |> filter(adnme6 == "Yes") |> 
dunn_test(formula = ICGR ~ Country, 
  p.adjust.method = "bonferroni", detailed = FALSE) |> flextable()
```

| .y. | group1 | group2 | n1 | n2 | statistic | p | p.adj | p.adj.signif |
| --- | --- | --- | --- | --- | --- | --- | --- | --- |
| ICGR | Ghana | Kenya | 290 | 619 | 3.049 | 0.002299 | 0.006898 | \*\* |
| ICGR | Ghana | Nigeria | 290 | 645 | 1.284 | 0.199103 | 0.597309 | ns |
| ICGR | Kenya | Nigeria | 619 | 645 | -2.242 | 0.024961 | 0.074884 | ns |

```
labelled_data |> filter(adnme6 == "Yes") |> 
dunn_test(formula = Prob_PGD ~ Country, 
  p.adjust.method = "bonferroni", detailed = FALSE) |> flextable()
```

| .y. | group1 | group2 | n1 | n2 | statistic | p | p.adj | p.adj.signif |
| --- | --- | --- | --- | --- | --- | --- | --- | --- |
| Prob\_PGD | Ghana | Kenya | 290 | 619 | 1.1848 | 0.2361 | 0.70834 | ns |
| Prob\_PGD | Ghana | Nigeria | 290 | 645 | -0.6515 | 0.5147 | 1.00000 | ns |
| Prob\_PGD | Kenya | Nigeria | 619 | 645 | -2.3170 | 0.0205 | 0.06151 | ns |

```
labelled_data |> filter(adnme6 == "Yes") |> 
dunn_test(formula = lost1y ~ Country, 
  p.adjust.method = "bonferroni", detailed = FALSE) |> flextable()
```

| .y. | group1 | group2 | n1 | n2 | statistic | p | p.adj | p.adj.signif |
| --- | --- | --- | --- | --- | --- | --- | --- | --- |
| lost1y | Ghana | Kenya | 290 | 619 | 2.958 | 0.003096 | 0.009289 | \*\* |
| lost1y | Ghana | Nigeria | 290 | 645 | 1.748 | 0.080479 | 0.241437 | ns |
| lost1y | Kenya | Nigeria | 619 | 645 | -1.545 | 0.122423 | 0.367270 | ns |

```
labelled_data |> filter(adnme6 == "Yes") |> 
dunn_test(formula = Educ ~ Country, 
  p.adjust.method = "bonferroni", detailed = FALSE) |> flextable()
```

| .y. | group1 | group2 | n1 | n2 | statistic | p | p.adj | p.adj.signif |
| --- | --- | --- | --- | --- | --- | --- | --- | --- |
| Educ | Ghana | Kenya | 290 | 619 | 1.876 | 0.060620 | 0.18186 | ns |
| Educ | Ghana | Nigeria | 290 | 645 | 3.174 | 0.001503 | 0.00451 | \*\* |
| Educ | Kenya | Nigeria | 619 | 645 | 1.615 | 0.106224 | 0.31867 | ns |

```
labelled_data |> filter(adnme6 == "Yes") |> 
dunn_test(formula = Age ~ Country, 
  p.adjust.method = "bonferroni", detailed = FALSE) |> flextable()
```

| .y. | group1 | group2 | n1 | n2 | statistic | p | p.adj | p.adj.signif |
| --- | --- | --- | --- | --- | --- | --- | --- | --- |
| Age | Ghana | Kenya | 290 | 619 | 1.669 | 0.095213761 | 0.285641282 | ns |
| Age | Ghana | Nigeria | 290 | 645 | 4.663 | 0.000003112 | 0.000009336 | \*\*\*\* |
| Age | Kenya | Nigeria | 619 | 645 | 3.749 | 0.000177216 | 0.000531649 | \*\*\* |

```
labelled_data |> filter(adnme6 == "Yes") |> 
wilcox_test(formula = ICGR_tot ~ Country, 
  p.adjust.method = "bonferroni", detailed = FALSE) |> flextable()
```

| .y. | group1 | group2 | n1 | n2 | statistic | p | p.adj | p.adj.signif |
| --- | --- | --- | --- | --- | --- | --- | --- | --- |
| ICGR\_tot | Ghana | Kenya | 290 | 619 | 77,806 | 0.001 | 0.004 | \*\* |
| ICGR\_tot | Ghana | Nigeria | 290 | 645 | 87,532 | 0.116 | 0.348 | ns |
| ICGR\_tot | Kenya | Nigeria | 619 | 645 | 214,108 | 0.025 | 0.076 | ns |

```
labelled_data |> filter(adnme6 == "Yes") |> 
  kruskal_test(formula = ICGR_tot ~ Country) |> flextable()
```

| .y. | n | statistic | df | p | method |
| --- | --- | --- | --- | --- | --- |
| ICGR\_tot | 1,554 | 11.7 | 2 | 0.00288 | Kruskal-Wallis |

```
cpal <- c("#edae49", "#d1495b", "#00798c")                                      # Consistent palette for Country plots.
comparisons <- list(c("Ghana","Kenya"),                                         # Specify `wilcox.test` comparisons for `ggpubr`
                    c("Ghana","Nigeria"), 
                    c("Kenya", "Nigeria"))

plotdata <- read.csv("Plotdata.csv")
plotdata <- plotdata |> filter(adnme6 == 1) |>                                  # Compute total for ICGR by pivoting row wise.
    rowwise() |> 
    mutate(ICGR_tot = sum(icgr1+icgr2+icgr3+icgr4+icgr5+icgr6+icgr7)) |> 
    ungroup()
#write.csv(plotdata, "Plotdata.csv")


stat_test <- plotdata |> wilcox_test(ICGR_tot ~ Country,                        # Manually set comparison for Figure 1 using `rstatix::wilcox_test`
 p.adjust.method = "bonferroni")                                                # using bonferroni adjustment for multiple comprehensions.

stat_test <- stat_test |>                                                       # Set x, y positions for `stat_test` in Figure 1.
 mutate(y.position = c(37,40,43),
        x = c("Ghana", "Kenya", "Nigeria"))


ggplot(plotdata, aes(Country, ICGR_tot)) +                                      # Base plot: Total Complex Grief Score by Country.
geom_violin(aes(fill = Country), alpha = .4) +                                  # Violin plot to represent underlying data distribution.           
  geom_boxplot(aes(fill = Country),                                             # Box plot to represent summary statistics.
               alpha = .5, width = .3, outlier.shape = NA) +                           
  ggpubr::stat_pvalue_manual(stat_test,                                         # Compare means between Country groups using `ggpubr`
                             method = "wilcox.test",                            # and `stat_test` specified above.
                             p.adjust.method = "bonferroni",                    
                             label = "p.adj.signif",
                             tip.length = 0.01) +               
  labs(x = NULL,                                                                # Labels and styling for Figure 1.        
       y = "Complex Grief Total Score", 
       title = NULL) +
  theme_minimal() + theme(legend.position = "none") +
  scale_color_manual(values = cpal) + 
  scale_fill_manual(values = cpal)
```

```
#  ggsave("PGD_Violinplot.jpeg", plot = last_plot(), width = 9,  height = 4,  units = "in",  dpi = 600)
```

### Network Estimation

```
PGDnet     <- estimateNetwork(PGD, default = "EBICglasso", corMethod = "cor", 
                              corArgs = list(method = "spearman"), tuning = 0.5)  # Use Spearman's Rank Correlation for non-normal data

#  Total sample plot and centrality
PGDnetplot <- plot(PGDnet, layout = "spring", negDashed = T, color = "ivory2", codenames = labels)
```

```
AfricaPlot <- qgraph(PGDnetplot, groups = subscales, color = c("#D6D84F", "#B9E28C", "ivory2"), legend = T,
                     edge.color = c("#44355B"),
                     legend.mode = "style1", legend.cex = 1.1, nodeNames = labels)
```

```
centralityPlot(PGDnet, 
               scale = "z-scores",
               include=c("Betweenness","Closeness", "Strength","ExpectedInfluence"), 
               orderBy = "ExpectedInfluence")
```

```
# ggsave("PGDnet_centrality.jpeg", plot = last_plot(), width = 9,  height = 4,  units = "in",  dpi = 600)


#  Sub-sample network estimation
GhanaNetEst      <- estimateNetwork(GhanaNet,   default = "EBICglasso", corMethod="cor", corArgs=list(method="spearman"), tuning = 0.5)
KenyaNetEst      <- estimateNetwork(KenyaNet,   default = "EBICglasso", corMethod="cor", corArgs=list(method="spearman"), tuning = 0.5)
NigeriaNetEst    <- estimateNetwork(NigeriaNet, default = "EBICglasso", corMethod="cor", corArgs=list(method="spearman"), tuning = 0.5)

# Print centrality incidences for all networks
centralityTable(PGDnet, GhanaNetEst, KenyaNetEst, NigeriaNetEst,
                standardized = TRUE,  relative = FALSE, 
                weighted = TRUE, signed = TRUE) |> pivot_wider(names_from = graph, values_from = value) |> 
                rename("Total" = "graph 1", "Ghana" = "graph 2", 
                       "Kenya" = "graph 3", "Nigeria" = "graph 4") |> flextable()
```

| type | node | measure | Total | Ghana | Kenya | Nigeria |
| --- | --- | --- | --- | --- | --- | --- |
|  | icgr1 | Betweenness | -0.38188 | 0.33813 | -0.9131 | -0.85863 |
|  | icgr2 | Betweenness | -1.14564 | -0.82118 | -0.9131 | -0.45457 |
|  | icgr3 | Betweenness | 0.38188 | 1.88388 | 1.0791 | 0.35355 |
|  | icgr4 | Betweenness | 0.38188 | -0.82118 | 0.4150 | -0.05051 |
|  | icgr5 | Betweenness | 1.90941 | 0.33813 | 1.7431 | 1.96980 |
|  | icgr6 | Betweenness | -0.38188 | -0.82118 | -0.2490 | -0.85863 |
|  | icgr7 | Betweenness | -1.14564 | -0.82118 | -0.2490 | -0.85863 |
|  | icgr8 | Betweenness | 0.38188 | 0.72457 | -0.9131 | 0.75761 |
|  | icgr1 | Closeness | 0.54261 | 0.89962 | -1.4942 | 0.50305 |
|  | icgr2 | Closeness | 0.07571 | -0.94166 | 0.1550 | 0.14176 |
|  | icgr3 | Closeness | 1.50488 | 1.10570 | 1.7251 | 1.49303 |
|  | icgr4 | Closeness | -0.19737 | -0.60118 | 0.2859 | 0.26438 |
|  | icgr5 | Closeness | 0.81149 | 0.89962 | 0.7154 | 0.55732 |
|  | icgr6 | Closeness | -0.61237 | 0.02143 | -0.1774 | -0.45364 |
|  | icgr7 | Closeness | -1.80579 | -1.67998 | -1.0580 | -1.87409 |
|  | icgr8 | Closeness | -0.31917 | 0.29645 | -0.1519 | -0.63182 |
|  | icgr1 | Strength | -0.04467 | 0.41337 | -0.8096 | 0.31816 |
|  | icgr2 | Strength | -0.99628 | -1.01971 | -1.0272 | -0.35664 |
|  | icgr3 | Strength | 1.85439 | 1.90492 | 1.5358 | 1.56020 |
|  | icgr4 | Strength | -0.02196 | -0.77307 | -0.1757 | 0.29212 |
|  | icgr5 | Strength | 0.90201 | 0.35379 | 1.5075 | 0.31623 |
|  | icgr6 | Strength | -0.84376 | -0.15210 | -0.1521 | -1.35309 |
|  | icgr7 | Strength | -1.00780 | -1.12197 | -0.7695 | -1.39331 |
|  | icgr8 | Strength | 0.15806 | 0.39477 | -0.1091 | 0.61633 |
|  | icgr1 | ExpectedInfluence | -0.04467 | 0.41337 | -0.8096 | 0.52750 |
|  | icgr2 | ExpectedInfluence | -0.99628 | -1.01971 | -1.0272 | -1.02001 |
|  | icgr3 | ExpectedInfluence | 1.85439 | 1.90492 | 1.5358 | 1.69610 |
|  | icgr4 | ExpectedInfluence | -0.02196 | -0.77307 | -0.1757 | 0.50300 |
|  | icgr5 | ExpectedInfluence | 0.90201 | 0.35379 | 1.5075 | 0.52568 |
|  | icgr6 | ExpectedInfluence | -0.84376 | -0.15210 | -0.1521 | -1.04493 |
|  | icgr7 | ExpectedInfluence | -1.00780 | -1.12197 | -0.7695 | -1.08277 |
|  | icgr8 | ExpectedInfluence | 0.15806 | 0.39477 | -0.1091 | -0.10457 |

```
par(mfrow = c(1,3))                                                             # Format plotting area to 1x3 to display network plots together.
#  Simple plots of sub-sample networks
GhanaNetPlot     <- plot(GhanaNetEst,   layout = "spring", negDashed = T, color = "#edae49", codenames = labels)
KenyaNetPlot     <- plot(KenyaNetEst,   layout = "spring", negDashed = T, color = "#d1495b", codenames = labels)
NigeriaNetPlot   <- plot(NigeriaNetEst, layout = "spring", negDashed = T, color = "#00798c", codenames = labels)
```

```
# `qgraph` plots with average layout for ease of comparison
AvLayout    <- averageLayout(GhanaNetPlot, KenyaNetPlot) 
GhanaPlot   <- qgraph(GhanaNetPlot,   layout = AvLayout, 
                      legend = FALSE, vsize = 12, color = "#edae49", 
                      posCol="#44355B", title = "Ghana", title.cex = 2)
KenyaPlot   <- qgraph(KenyaNetPlot,   layout = AvLayout, 
                      legend = FALSE, vsize = 12, color = "#d1495b", 
                      posCol="#44355B", title = "Kenya", title.cex = 2)
NigeriaPlot <- qgraph(NigeriaNetPlot, layout = AvLayout, 
                      legend = FALSE, vsize = 12, color = "#00798c", 
                      posCol="#44355B", title = "Nigeria", title.cex = 2)
```

```
## Figure 1
#  jpeg("Network_plots.jpeg", width = 1200, height = 900)
layout(matrix(c(1,1,1,
                2,3,4), nrow=2, byrow = TRUE))
qgraph(AfricaPlot, title = "Total Sample", title.cex = 3)
qgraph(GhanaPlot)
qgraph(KenyaPlot)
qgraph(NigeriaPlot)
```

```
#  dev.off()

par(mfrow=c(1,1))                                                               # Return to 1x1 plotting.

# Inspect edge weights
qgraph(GhanaPlot, edge.labels = TRUE)
```

```
qgraph(KenyaPlot, edge.labels = TRUE)
```

```
qgraph(NigeriaPlot, edge.labels = TRUE)
```

Correlation matrices and assumption checks of ICGR variables were
inspected (`base R` | `ggplot2`) and Networks
estimated (`bootnet`).

```
options(scipen = 999, digits = 4)                                               # Remove scientific notation and set sig figures for tables.

#  Descriptives for ICGR data
psych::describe(PGD)        |> flextable()
```

| vars | n | mean | sd | median | trimmed | mad | min | max | range | skew | kurtosis | se |
| --- | --- | --- | --- | --- | --- | --- | --- | --- | --- | --- | --- | --- |
| 1 | 1,554 | 2.499 | 1.1838 | 2 | 2.406 | 1.483 | 1 | 5 | 4 | 0.4243 | -0.5976 | 0.03003 |
| 2 | 1,554 | 2.954 | 1.2738 | 3 | 2.943 | 1.483 | 1 | 5 | 4 | 0.1994 | -1.0101 | 0.03231 |
| 3 | 1,554 | 2.380 | 1.2535 | 2 | 2.255 | 1.483 | 1 | 5 | 4 | 0.5317 | -0.7306 | 0.03180 |
| 4 | 1,554 | 2.559 | 1.2917 | 3 | 2.451 | 1.483 | 1 | 5 | 4 | 0.3581 | -0.9356 | 0.03277 |
| 5 | 1,554 | 1.914 | 1.0898 | 2 | 1.738 | 1.483 | 1 | 5 | 4 | 1.0063 | 0.1422 | 0.02765 |
| 6 | 1,554 | 2.624 | 1.3615 | 2 | 2.530 | 1.483 | 1 | 5 | 4 | 0.4256 | -1.0379 | 0.03454 |
| 7 | 1,554 | 1.631 | 1.0872 | 1 | 1.378 | 0.000 | 1 | 5 | 4 | 1.7573 | 2.1494 | 0.02758 |
| 8 | 1,554 | 1.665 | 0.9756 | 1 | 1.479 | 0.000 | 1 | 5 | 4 | 1.4652 | 1.4767 | 0.02475 |

```
psych::describe(GhanaNet)   |> flextable()
```

| vars | n | mean | sd | median | trimmed | mad | min | max | range | skew | kurtosis | se |
| --- | --- | --- | --- | --- | --- | --- | --- | --- | --- | --- | --- | --- |
| 1 | 290 | 2.483 | 1.2401 | 2 | 2.366 | 1.483 | 1 | 5 | 4 | 0.4407 | -0.6841 | 0.07282 |
| 2 | 290 | 2.786 | 1.2871 | 3 | 2.733 | 1.483 | 1 | 5 | 4 | 0.3136 | -0.9067 | 0.07558 |
| 3 | 290 | 2.307 | 1.2694 | 2 | 2.168 | 1.483 | 1 | 5 | 4 | 0.5965 | -0.7412 | 0.07454 |
| 4 | 290 | 2.424 | 1.2682 | 2 | 2.328 | 1.483 | 1 | 5 | 4 | 0.3675 | -1.0205 | 0.07447 |
| 5 | 290 | 1.855 | 1.0554 | 1 | 1.685 | 0.000 | 1 | 5 | 4 | 1.0464 | 0.2072 | 0.06198 |
| 6 | 290 | 2.310 | 1.2562 | 2 | 2.155 | 1.483 | 1 | 5 | 4 | 0.7388 | -0.4709 | 0.07377 |
| 7 | 290 | 1.524 | 1.0228 | 1 | 1.263 | 0.000 | 1 | 5 | 4 | 2.0331 | 3.2109 | 0.06006 |
| 8 | 290 | 1.572 | 0.9356 | 1 | 1.371 | 0.000 | 1 | 5 | 4 | 1.7481 | 2.5482 | 0.05494 |

```
psych::describe(KenyaNet)   |> flextable()
```

| vars | n | mean | sd | median | trimmed | mad | min | max | range | skew | kurtosis | se |
| --- | --- | --- | --- | --- | --- | --- | --- | --- | --- | --- | --- | --- |
| 1 | 619 | 2.562 | 1.128 | 3 | 2.497 | 1.483 | 1 | 5 | 4 | 0.3621 | -0.5201 | 0.04534 |
| 2 | 619 | 3.120 | 1.273 | 3 | 3.139 | 1.483 | 1 | 5 | 4 | 0.1049 | -1.1262 | 0.05116 |
| 3 | 619 | 2.480 | 1.258 | 2 | 2.368 | 1.483 | 1 | 5 | 4 | 0.4542 | -0.8003 | 0.05058 |
| 4 | 619 | 2.695 | 1.283 | 3 | 2.620 | 1.483 | 1 | 5 | 4 | 0.2650 | -0.9397 | 0.05157 |
| 5 | 619 | 2.074 | 1.158 | 2 | 1.922 | 1.483 | 1 | 5 | 4 | 0.7732 | -0.3725 | 0.04654 |
| 6 | 619 | 2.519 | 1.334 | 2 | 2.400 | 1.483 | 1 | 5 | 4 | 0.5211 | -0.9010 | 0.05364 |
| 7 | 619 | 1.806 | 1.187 | 1 | 1.567 | 0.000 | 1 | 5 | 4 | 1.3919 | 0.8493 | 0.04771 |
| 8 | 619 | 1.832 | 1.068 | 1 | 1.642 | 0.000 | 1 | 5 | 4 | 1.1813 | 0.5506 | 0.04292 |

```
psych::describe(NigeriaNet) |> flextable()
```

| vars | n | mean | sd | median | trimmed | mad | min | max | range | skew | kurtosis | se |
| --- | --- | --- | --- | --- | --- | --- | --- | --- | --- | --- | --- | --- |
| 1 | 645 | 2.445 | 1.2090 | 2 | 2.337 | 1.483 | 1 | 5 | 4 | 0.4840 | -0.6228 | 0.04760 |
| 2 | 645 | 2.871 | 1.2525 | 3 | 2.839 | 1.483 | 1 | 5 | 4 | 0.2433 | -0.9293 | 0.04932 |
| 3 | 645 | 2.318 | 1.2373 | 2 | 2.186 | 1.483 | 1 | 5 | 4 | 0.5798 | -0.6484 | 0.04872 |
| 4 | 645 | 2.488 | 1.3005 | 2 | 2.364 | 1.483 | 1 | 5 | 4 | 0.4484 | -0.8855 | 0.05121 |
| 5 | 645 | 1.786 | 1.0173 | 1 | 1.613 | 0.000 | 1 | 5 | 4 | 1.2313 | 0.8262 | 0.04006 |
| 6 | 645 | 2.865 | 1.3928 | 3 | 2.832 | 1.483 | 1 | 5 | 4 | 0.1998 | -1.2431 | 0.05484 |
| 7 | 645 | 1.512 | 0.9900 | 1 | 1.273 | 0.000 | 1 | 5 | 4 | 2.0819 | 3.6665 | 0.03898 |
| 8 | 645 | 1.547 | 0.8739 | 1 | 1.373 | 0.000 | 1 | 5 | 4 | 1.6257 | 2.1343 | 0.03441 |

```
#  Print Correlation Matrices
cor(PGD,        use = "complete.obs") |> as.data.frame() |>  flextable()
```

| icgr1 | icgr2 | icgr3 | icgr4 | icgr5 | icgr6 | icgr7 | icgr8 |
| --- | --- | --- | --- | --- | --- | --- | --- |
| 1.0000 | 0.5485 | 0.6744 | 0.6127 | 0.6093 | 0.4881 | 0.4506 | 0.5170 |
| 0.5485 | 1.0000 | 0.6219 | 0.5176 | 0.5543 | 0.4969 | 0.4137 | 0.4302 |
| 0.6744 | 0.6219 | 1.0000 | 0.6613 | 0.6443 | 0.5359 | 0.5225 | 0.5701 |
| 0.6127 | 0.5176 | 0.6613 | 1.0000 | 0.5397 | 0.5385 | 0.4393 | 0.4857 |
| 0.6093 | 0.5543 | 0.6443 | 0.5397 | 1.0000 | 0.5245 | 0.6019 | 0.6718 |
| 0.4881 | 0.4969 | 0.5359 | 0.5385 | 0.5245 | 1.0000 | 0.4895 | 0.4907 |
| 0.4506 | 0.4137 | 0.5225 | 0.4393 | 0.6019 | 0.4895 | 1.0000 | 0.6370 |
| 0.5170 | 0.4302 | 0.5701 | 0.4857 | 0.6718 | 0.4907 | 0.6370 | 1.0000 |

```
cor(GhanaNet,   use = "complete.obs") |> as.data.frame() |>  flextable()
```

| icgr1 | icgr2 | icgr3 | icgr4 | icgr5 | icgr6 | icgr7 | icgr8 |
| --- | --- | --- | --- | --- | --- | --- | --- |
| 1.0000 | 0.5765 | 0.6947 | 0.5976 | 0.6352 | 0.5610 | 0.4191 | 0.5483 |
| 0.5765 | 1.0000 | 0.6291 | 0.4734 | 0.4611 | 0.4970 | 0.3588 | 0.4123 |
| 0.6947 | 0.6291 | 1.0000 | 0.6733 | 0.6196 | 0.6214 | 0.4753 | 0.5712 |
| 0.5976 | 0.4734 | 0.6733 | 1.0000 | 0.4804 | 0.4774 | 0.3055 | 0.4362 |
| 0.6352 | 0.4611 | 0.6196 | 0.4804 | 1.0000 | 0.5925 | 0.5578 | 0.6765 |
| 0.5610 | 0.4970 | 0.6214 | 0.4774 | 0.5925 | 1.0000 | 0.4951 | 0.6049 |
| 0.4191 | 0.3588 | 0.4753 | 0.3055 | 0.5578 | 0.4951 | 1.0000 | 0.5749 |
| 0.5483 | 0.4123 | 0.5712 | 0.4362 | 0.6765 | 0.6049 | 0.5749 | 1.0000 |

```
cor(KenyaNet,   use = "complete.obs") |> as.data.frame() |>  flextable()
```

| icgr1 | icgr2 | icgr3 | icgr4 | icgr5 | icgr6 | icgr7 | icgr8 |
| --- | --- | --- | --- | --- | --- | --- | --- |
| 1.0000 | 0.5358 | 0.6601 | 0.5940 | 0.6283 | 0.5262 | 0.4936 | 0.5259 |
| 0.5358 | 1.0000 | 0.6289 | 0.5446 | 0.6275 | 0.5522 | 0.4909 | 0.5101 |
| 0.6601 | 0.6289 | 1.0000 | 0.6782 | 0.6874 | 0.5782 | 0.5867 | 0.6129 |
| 0.5940 | 0.5446 | 0.6782 | 1.0000 | 0.6024 | 0.5964 | 0.4976 | 0.5188 |
| 0.6283 | 0.6275 | 0.6874 | 0.6024 | 1.0000 | 0.6222 | 0.6627 | 0.7038 |
| 0.5262 | 0.5522 | 0.5782 | 0.5964 | 0.6222 | 1.0000 | 0.6101 | 0.5757 |
| 0.4936 | 0.4909 | 0.5867 | 0.4976 | 0.6627 | 0.6101 | 1.0000 | 0.6726 |
| 0.5259 | 0.5101 | 0.6129 | 0.5188 | 0.7038 | 0.5757 | 0.6726 | 1.0000 |

```
cor(NigeriaNet, use = "complete.obs") |> as.data.frame() |>  flextable()
```

| icgr1 | icgr2 | icgr3 | icgr4 | icgr5 | icgr6 | icgr7 | icgr8 |
| --- | --- | --- | --- | --- | --- | --- | --- |
| 1.0000 | 0.5465 | 0.6776 | 0.6359 | 0.5838 | 0.4525 | 0.4245 | 0.5011 |
| 0.5465 | 1.0000 | 0.6066 | 0.5010 | 0.5073 | 0.4760 | 0.3324 | 0.3269 |
| 0.6776 | 0.6066 | 1.0000 | 0.6359 | 0.6068 | 0.4899 | 0.4667 | 0.5182 |
| 0.6359 | 0.5010 | 0.6359 | 1.0000 | 0.4922 | 0.5379 | 0.4231 | 0.4626 |
| 0.5838 | 0.5073 | 0.6068 | 0.4922 | 1.0000 | 0.4519 | 0.5267 | 0.6123 |
| 0.4525 | 0.4760 | 0.4899 | 0.5379 | 0.4519 | 1.0000 | 0.4105 | 0.4090 |
| 0.4245 | 0.3324 | 0.4667 | 0.4231 | 0.5267 | 0.4105 | 1.0000 | 0.5984 |
| 0.5011 | 0.3269 | 0.5182 | 0.4626 | 0.6123 | 0.4090 | 0.5984 | 1.0000 |

```
#  Correlation Matrix Plot
## Function to grab values for corr matrix
get_upper_tri <- function(cormat){
    cormat[lower.tri(cormat)]<- NA
    return(cormat)
}

upper_tri <- get_upper_tri(round(cor(PGD), 2))                                  # Required correlations for bereaved sample on ICGR items.

## Correlation Matrix Figure
ggplot(data = reshape2::melt(upper_tri, na.rm = TRUE), 
       aes(Var2, Var1, fill = value))+
 geom_tile(color = "white") +
 geom_text(aes(Var2, Var1, label = value), color = "black", size = 4) +
 scale_fill_gradient2(low = "#175676", high = "#ba324f", mid = "white", 
   midpoint = 0, limit = c(-1,1), space = "Lab", 
   name="Correlation") +
  theme_minimal() + 
  theme(axis.title.x = element_blank(),
  axis.title.y = element_blank(),
  panel.grid.major = element_blank(),
  panel.border = element_blank(),
  panel.background = element_blank(),
  axis.ticks = element_blank(),
  legend.justification = c(1, 0),
  legend.position = c(0.3, 0.7),
  legend.direction = "horizontal")+
  guides(fill = guide_colorbar(barwidth = 7, barheight = 1,
                title.position = "top", title.hjust = 0.5))
```

```
 coord_fixed()
```

```
## <ggproto object: Class CoordFixed, CoordCartesian, Coord, gg>
##     aspect: function
##     backtransform_range: function
##     clip: on
##     default: FALSE
##     distance: function
##     expand: TRUE
##     is_free: function
##     is_linear: function
##     labels: function
##     limits: list
##     modify_scales: function
##     range: function
##     ratio: 1
##     render_axis_h: function
##     render_axis_v: function
##     render_bg: function
##     render_fg: function
##     setup_data: function
##     setup_layout: function
##     setup_panel_guides: function
##     setup_panel_params: function
##     setup_params: function
##     train_panel_guides: function
##     transform: function
##     super:  <ggproto object: Class CoordFixed, CoordCartesian, Coord, gg>
```

```
#  Assumption Checks for Network Analysis
networktools::assumptionCheck(PGD,
type = c("network", "impact"),
percent = 20,
split = c("median", "mean", "forceEqual", "cutEqual", "quartiles"),
plot = TRUE,
binary.data = FALSE,
na.rm = TRUE)
```

```
## Grand mean of variances =  1.43 
## Node variances 
##  icgr1  icgr2  icgr3  icgr4  icgr5  icgr6  icgr7  icgr8 
## 1.4015 1.6225 1.5713 1.6685 1.1877 1.8537 1.1820 0.9517 
## 
##  Shapiro-Wilk Normality (p) 
##                                                  icgr1                                                  icgr2                                                  icgr3 
## 0.0000000000000000000000000000001102380207571523077544 0.0000000000000000000000000000019291448611201633133588 0.0000000000000000000000000000000004070708029238313015 
##                                                  icgr4                                                  icgr5                                                  icgr6 
## 0.0000000000000000000000000000000245898335272523053149 0.0000000000000000000000000000000000000000920435835534 0.0000000000000000000000000000000018985962315686697836 
##                                                  icgr7                                                  icgr8 
## 0.0000000000000000000000000000000000000000000000002564 0.0000000000000000000000000000000000000000000008585618
```

### Network Comparison Test

Compare centrality function originally authored by R.R.
Gabriel. Iterated for comparison of three networks based on
theoretical perspectives authored by van Borkulo et al.

View Function Scripts

```
compareCentrality <- function(net1, net2,
                              include = c("Strength",
                                          "Closeness",
                                          "Betweenness",
                                          "ExpectedInfluence",
                                          "all",
                                          "All"),
                              orderBy = c("Strength",
                                          "Closeness",
                                          "Betweenness",
                                          "ExpectedInfluence"),
                              decreasing = T,
                              legendName = c("Strength",
                                          "Closeness",
                                          "Betweenness",
                                          "Exp'd Influence"),
                              net1Name = 'Network 1',
                              net2Name = 'Network 2'){
  
  library(ggplot2)
  library(forcats)
  
  if(include == "All" | include == "all"){
     include = c("Strength",
                "Closeness",
                "Betweenness",
                "ExpectedInfluence")
  }
  
  df <- centralityTable(net1, net2) %>% filter(measure %in% include)
  
  df %>% 
    mutate(graph = case_when(graph == 'graph 1' ~ net1Name,
                             graph == 'graph 2' ~ net2Name),
           graph = as.factor(graph),
           node = as.factor(node)) %>% 
           mutate(node = fct_reorder(node, value)) %>% 
    
    ggplot(aes(x = node, y = value, group = graph)) +
    geom_line(aes(color = graph), size = 1) + scale_color_manual(values = c("#edae49", "#d1495b", "#00798c")) +
    labs(x = '', y = '') +
    scale_linetype_discrete(name = legendName) +
    coord_flip() +
    facet_grid(~measure) +
    theme_bw()
  
}

difference_value <- function(NCT, alpha = 0.05){
  
  diff_edges <- NCT$einv.pvals %>% dplyr::filter(`p-value` <= alpha)
  
  for (i in 1:nrow(diff_edges)) {
    var_1 <- as.character(diff_edges[i, 1])
    var_2 <- as.character(diff_edges[i, 2])
    
    value_net_1 <- NCT$nw1[var_1, var_2]
    value_net_2 <- NCT$nw2[var_1, var_2]
    
    abs_difference <- abs(value_net_1 - value_net_2)
    p_value <- diff_edges$`p-value`[i]
    
    cat("Test Edge", i, "\n----\n")
    cat(var_1, "and", var_2)
    cat("\nNetwork 1:", value_net_1,
        "\nNetwork 2:", value_net_2)
    cat("\nAbsolute difference:", abs_difference,
        "with p-value =", p_value, "\n----\n")
  }
}
```

```
#  Credit: https://reisrgabriel.com/blog/2021-10-11-compare-centrality/

requiredPackages <- c("tidyverse", "forcats",                                   # Select and load required packaged for function
                      "qgraph", "NetworkComparisonTest")   
lapply(requiredPackages, require, character.only = TRUE)
```

```
## [[1]]
## [1] TRUE
## 
## [[2]]
## [1] TRUE
## 
## [[3]]
## [1] TRUE
## 
## [[4]]
## [1] TRUE
```

```
compareCentrality <- function(net1, net2, net3,                                 # `net1`:`net3` should be estimated networks 
                              include = c("Strength",                           # `net1` <- bootnet::estimateNetwork()
                                          "Closeness",
                                          "Betweenness",
                                          "ExpectedInfluence",
                                          "all",
                                          "All"),
                              orderBy = c("Strength",                           # Set display order for figure (L to R).
                                          "Closeness",
                                          "Betweenness",
                                          "ExpectedInfluence"),
                              decreasing = TRUE,                                # Arrange nodes decreasing in Strength
                              legendName = c("Strength",
                                          "Closeness",
                                          "Betweenness",
                                          "Exp'd Influence"),
                              net1Name = 'Network 1',                           # Function to read network names from centrality table.
                              net2Name = 'Network 2',
                              net3Name = 'Network 3'){

  
  if(include == "All" | include == "all"){
    include = c("ExpectedInfluence",
                "Strength",
                "Closeness",
                "Betweenness")
  }
  
  df <- qgraph::centralityTable(net1, net2, net3,                               # Load centalityTable to `df` for plotting
                standardized = TRUE,  relative = FALSE, 
                weighted = TRUE, signed = TRUE) |> filter(measure %in% include)
  
  df |> 
    mutate(graph = case_when(graph == 'graph 1' ~ net1Name,                     # transform variables in `df`
                             graph == 'graph 2' ~ net2Name,
                             graph == 'graph 3' ~ net3Name),
           Network = as.factor(graph),                                          # Factorise the nodes and edge weights to read to `ggplot`
           node = as.factor(node))  |>  
           mutate(node = fct_reorder(node, value))  |>  
    
    ggplot(aes(x = node, y = value, group = Network)) +
    geom_line(aes(colour = Network), size = 1) + 
    scale_color_manual(values = c("#edae49", "#d1495b", "#00798c")) +           # Manually set colour palette with values n=graphs.
    labs(x = '', y = '') +
    scale_linetype_discrete(name = legendName) +
    coord_flip() +
    facet_grid(~measure) +                                                      # Use `facet_grid` to display all centrality metrics in one figure
    theme_bw()
  
}

difference_value <- function(NCT, alpha = 0.05){                                # `difference_value` function 
  
  diff_edges <- NCT$einv.pvals |>  dplyr::filter('p-value' <= alpha)
  
  for (i in 1:nrow(diff_edges)) {
    var_1 <- as.character(diff_edges[i, 1])
    var_2 <- as.character(diff_edges[i, 2])
    var_3 <- as.character(diff_edges[i, 3])
    
    value_net_1 <- NCT$nw1[var_1, var_2]
    value_net_2 <- NCT$nw2[var_1, var_2]
    value_net_3 <- NCT$nw3[var_1, var_2]
    
    abs_difference <- abs(value_net_1 - value_net_2)
    p_value        <- diff_edges$`p-value`[i]
    
    cat("Test Edge", i, "\n----\n")
    cat(var_1, "and", var_2)
    cat("\nNetwork 1:", value_net_1,
        "\nNetwork 2:", value_net_2,
        "\nNetwork 3:", value_net_3)
    cat("\nAbsolute difference:", abs_difference,
        "with p-value =", p_value, "\n----\n")
  }
}
```

Differences between the three groups on structure and strength
invariance are shown in the `flextable` below.

```
#  Network Comparison Test using NCT, iterations to be set to 1000.
netcomp1 <- NCT(GhanaNetEst, KenyaNetEst , it = 1000, binary.data = FALSE, 
               test.edges = FALSE, edges = "all", progressbar = FALSE)
netcomp2 <- NCT(GhanaNetEst, NigeriaNetEst, it = 1000, binary.data = FALSE, 
               test.edges = FALSE, edges = "all", progressbar = FALSE)
netcomp3 <- NCT(NigeriaNetEst, KenyaNetEst , it = 1000, binary.data = FALSE, 
               test.edges = FALSE, edges = "all", progressbar = FALSE)

#  Plot Three-way Network Comparison  
CentralityCompPlot <- compareCentrality(GhanaNetEst, KenyaNetEst, NigeriaNetEst,
                        include    = "all",
                        orderBy    = "ExpectedInfluence",
                        legendName = "Network Centrality Comparison",
                        net1Name   = "Ghana",
                        net2Name   = "Kenya",
                        net3Name   = "Nigeria")
CentralityCompPlot
```

```
# ggsave("Centrality_Comparison.jpeg", plot = CentralityCompPlot, width = 9,  height = 4,  units = "in",  dpi = 600)


NetComparsionTable <- 
  data.frame(Network1 = c("Ghana", "Kenya", "Nigeria"),
             Network2 = c("Kenya", "Nigeria", "Kenya"),
             Network_Invariance_M = c(1,2,3),
             p_val_M = c(1,2,3),
             Global_Strength_S = c(1,2,3),
             p_val_S = c(1,2,3)
             ) 

NetComparsionTable[1,3] <- netcomp1$nwinv.real
NetComparsionTable[2,3] <- netcomp2$nwinv.real
NetComparsionTable[3,3] <- netcomp3$nwinv.real

NetComparsionTable[1,4] <- netcomp1$nwinv.pval
NetComparsionTable[2,4] <- netcomp2$nwinv.pval
NetComparsionTable[3,4] <- netcomp3$nwinv.pval

NetComparsionTable[1,5] <- netcomp1$glstrinv.real
NetComparsionTable[2,5] <- netcomp2$glstrinv.real
NetComparsionTable[3,5] <- netcomp3$glstrinv.real

NetComparsionTable[1,6] <- netcomp1$glstrinv.pval
NetComparsionTable[2,6] <- netcomp2$glstrinv.pval
NetComparsionTable[3,6] <- netcomp3$glstrinv.pval

NetComparsionTable |> flextable() # |> save_as_docx(path = "Table2.docx")
```

| Network1 | Network2 | Network\_Invariance\_M | p\_val\_M | Global\_Strength\_S | p\_val\_S |
| --- | --- | --- | --- | --- | --- |
| Ghana | Kenya | 0.2247 | 0.052 | 0.11745 | 0.089 |
| Kenya | Nigeria | 0.2285 | 0.031 | 0.14209 | 0.426 |
| Nigeria | Kenya | 0.1191 | 0.598 | 0.02464 | 0.695 |

### Total Sample Network Stability

For corStability Epskamp et al. (2018) suggest *“the
CS-coefficient should not be below 0.25, and preferably above 0.5.”*
The results below indicate that solid interpretations may be made based
on the total sample network owing to favourable centrality across
indices.

```
edgestab_TotalNetq <- bootnet(PGDnet, 
                              nBoots = 1000, # number of boot samples update to 1000
                              nCores = 2)
plot(edgestab_TotalNetq,
     labels = FALSE,
     order = "sample")
```

```
censtab_TotalNetq <- bootnet(PGDnet, 
                             nBoots = 1000, # number of boot samples update to 1000
                             type = "case",
                             nCores = 2,
                             statistics = c('strength',
                                            'expectedInfluence',
                                            'betweenness',
                                            'closeness'))


plot(censtab_TotalNetq, "all")
```

```
corStability(censtab_TotalNetq, cor = 0.7, statistics = "all", verbose = TRUE)
```

```
## === Correlation Stability Analysis === 
## 
## Sampling levels tested:
##    nPerson Drop%   n
## 1      388  75.0  89
## 2      509  67.2  94
## 3      630  59.5 109
## 4      751  51.7 101
## 5      872  43.9 102
## 6      993  36.1 105
## 7     1114  28.3 100
## 8     1235  20.5  95
## 9     1355  12.8  90
## 10    1476   5.0 115
## 
## Maximum drop proportions to retain correlation of 0.7 in at least 95% of the samples:
## 
## betweenness: 0.283 
##   - For more accuracy, run bootnet(..., caseMin = 0.205, caseMax = 0.361) 
## 
## closeness: 0.672 
##   - For more accuracy, run bootnet(..., caseMin = 0.595, caseMax = 0.75) 
## 
## expectedInfluence: 0.75 (CS-coefficient is highest level tested)
##   - For more accuracy, run bootnet(..., caseMin = 0.672, caseMax = 1) 
## 
## strength: 0.75 (CS-coefficient is highest level tested)
##   - For more accuracy, run bootnet(..., caseMin = 0.672, caseMax = 1) 
## 
## Accuracy can also be increased by increasing both 'nBoots' and 'caseN'.
```
