# Supplementary material for: Prolonged grief disorder symptomology in three African countries: A network analysis and comparison
Source: Glob Ment Health (Camb). 2024 Apr 29;11:e57. doi: 10.1017/gmh.2024.54 (PMC11094551; doi:10.1017/gmh.2024.54)
Supplement: Peer review history

# Editor report: Recommendation: Prolonged grief disorder symptomology in three African countries: A network analysis and comparison — R0/PR2

**DOI:** 10.1017/gmh.2024.54.pr2

**Affiliations:** Primary Healthcare Sciences, University of Zimbabwe Faculty of Medicine, Zimbabwe

No accompanying comment.

# Editor report: Decision: Prolonged grief disorder symptomology in three African countries: A network analysis and comparison — R0/PR3

**DOI:** 10.1017/gmh.2024.54.pr3

**Affiliations:** London School of Hygiene & Tropical Medicine, United Kingdom of Great Britain and Northern Ireland

No accompanying comment.

# Editor report: Recommendation: Prolonged grief disorder symptomology in three African countries: A network analysis and comparison — R1/PR5

**DOI:** 10.1017/gmh.2024.54.pr5

**Affiliations:** Primary Healthcare Sciences, University of Zimbabwe Faculty of Medicine, Zimbabwe

Kindly address the conceptual framework, particularly using consistent terminology.

# Editor report: Decision: Prolonged grief disorder symptomology in three African countries: A network analysis and comparison — R1/PR6

**DOI:** 10.1017/gmh.2024.54.pr6

**Affiliations:** London School of Hygiene & Tropical Medicine, United Kingdom of Great Britain and Northern Ireland

No accompanying comment.

# Editor report: Recommendation: Prolonged grief disorder symptomology in three African countries: A network analysis and comparison — R2/PR8

**DOI:** 10.1017/gmh.2024.54.pr8

**Affiliations:** Primary Healthcare Sciences, University of Zimbabwe Faculty of Medicine, Zimbabwe

No accompanying comment.

# Editor report: Decision: Prolonged grief disorder symptomology in three African countries: A network analysis and comparison — R2/PR9

**DOI:** 10.1017/gmh.2024.54.pr9

**Affiliations:** London School of Hygiene & Tropical Medicine, United Kingdom of Great Britain and Northern Ireland

No accompanying comment.
